# CDO1 is a new biomarker to discriminate aggressive forms of prostate cancer

**DOI:** 10.1038/s41388-026-03842-5

**Published:** 2026-06-09

**Authors:** Jeanne Gaspar Lopes, Angel Markovic, Cécile M Champy, Nicolas Aubourg, Jacques RR Mathieu, Federica Alberti, Pascale Soyeux-Porte, Kadiatou Drame, Marine Louarn, Thibaud Jamet, Romain Huc, Alexandre de la Taille, Charles-Antoine Dutertre, Damien Destouches, Francis Vacherot, Virginie Firlej

**Affiliations:** 1 TRePCa, Univ Paris Est Creteil, Creteil, France; 2 Institut Mondor de recherche biomédicale (IMRB), Univ Paris Est Creteil, Inserm U955, Créteil, France; 3 Department of Urology, AP-HP, Centre Hospitalier Universitaire Henri Mondor, Créteil, France; 4 INSERM U1279, Gustave Roussy, Villejuif, France; Université Paris Saclay, Villejuif, France; 5 Department of Pathology, AP-HP, Centre Hospitalier Universitaire Henri Mondor, Créteil, France; 6 Experimental and Translational Pathology Platform, AMMICa, INSERM US23/UAR3655, Gustave Roussy, Université Paris Saclay, Villejuif, France

**Keywords:** Prostate cancer, Tumour biomarkers

## Abstract

Worldwide, prostate cancer (PCa) ranks second in terms of incidence and eighth in terms of mortality. While most cancers remain silent after initial treatment, some tumors recur. At present, we are unable to predict which patients are at risk of recurrence. In order to determine this risk of recurrence as early as at the biopsy stage, and to enable better therapeutic management of patients, it is essential to identify new biomarkers. In this study, we have demonstrated that Cysteine Dioxygenase CDO1 could be a predictive marker in PCa progression. Transcriptomic analysis showed that CDO1 expression is significantly reduced in patients who have relapsed and lower CDO1 expression is associated with poorer survival outcomes. In PCa, CDO1 expression could be regulated by methylation and androgen signaling pathway. Furthermore, inhibition of CDO1 expression in VCaP prostate cancer cells led to increased cell migration and non-adherent growth. Finally, transcriptomic analysis of these cells with inhibited CDO1 expression demonstrates the complex role of CDO1 and its possible involvement in the mechanisms regulating endoplasmic reticulum stress and protein unfolding. Altogether, these results describe the loss of CDO1 as a marker of aggressiveness in PCa. Furthermore, the loss of CDO1 is thought to be responsible for tumor progression in vitro by acting on multiple signaling pathways.

## Introduction

Prostate cancer (PCa) is worldwide the second leading cancer in men and ranks eighth in terms of mortality [1]. While most PCa progress favorably after radiotherapy or radical surgery, 20% of patients will experience recurrence. At this stage, patients will be treated by androgen deprivation therapy (ADT) in combination or not with second-generation hormone therapies. However, resistance to these treatments inevitably develops, leading to metastatic disease and ultimately a fatal outcome. Currently, there is no early test available to guide treatment. Therapeutic strategies are primarily based on the ISUP score, MRI, and PSA level. However, in some cases, patient outcome seems to be independent of the stage and/or the Gleason score which indicates the degree of aggressiveness of the cancer. Thus, it is still impossible to distinguish relapsing and non-relapsing disease. Precise and effective markers would therefore enable us to differentiate those two forms, to better adapt management and avoid any over-treatment that could lead to significant side effects.

Cysteine dioxygenase type 1 (CDO1) is a cytosolic enzyme involved in taurine biosynthesis and in the regulation of cysteine concentration [2]. In physiological condition, this non-heme iron metalloenzyme catalyses the reaction of L-cysteine to cysteine-sulfonic acid (CSA) in the presence of oxygen. CSA can then be converted to hypotaurine to form taurine, or to 3-sulfinylpyruvate to form pyruvate preventing from cysteine accumulation. Defective CDO1 activity is associated with cytotoxicity and neurotoxicity and plays a role in inflammatory (rheumatoid arthritis) and neurodegenerative (Parkinson’s and Alzheimer’s disease) diseases [3]. CDO-/- mice present elevated cysteine levels. In this case, cysteine is metabolized via CDO1-independent pathways. Nevertheless, an excessive increase in H2S/HS- production is observed in tissues, leading to cytotoxicity, particularly in the pancreas and lungs [4, 5]. Abnormal expression of CDO1 mRNA has been observed in several cancers and the vast majority of these involve a decrease in its expression, linked to epigenetic silencing by methylation of its promoter (colorectal, gastric, breast, lung, bladder) [6]. In PCa, studies have shown hypermethylation of the CDO1 promoter in different cancer cell lines compared to healthy cell lines associated with a decrease in CDO1 expression [7]. In cancer cells, CDO1 loss has been shown to elevate intracellular cysteine, reduce cystine uptake, and preserve NADPH, which raises the NADPH/NADP⁺ ratio, therefore enhancing antioxidant defenses which support tumor growth. The excess cysteine also increases GSH production, increasing GPX4 activity and suppressing ferroptosis [8], thereby improving cancer cell survival. With strengthened ROS‑detoxifying capacity, cancer cells become more resistant to oxidative stress and less sensitive to treatments such as anthracyclines [9]. In addition, CDO1 silencing increases H₂S levels, which amplifies the Warburg effect and contributes to mitochondrial dysfunction [10]. Altogether, the metabolic and redox rewiring triggered by CDO1 loss promotes cancer cell proliferation, therapy resistance, and tumorigenesis.

However, few information is available regarding the relationship between CDO1 and tumor growth. In this study, we showed that CDO1 might be an interesting marker in aggressive forms of PCa. We demonstrated particularly its role in tumor proliferation and its potential regulation by AR signaling. Our results suggest that CDO1 expression is positively regulated by AR and that decreased CDO1 expression in tumor cells is correlated with prostate cancer aggressiveness. Finally, our work highlights that CDO1 loss in aggressive PCa could play a role as a protection against Unfolded Protein Response and endoplasmic reticulum stress responses.

## Material and methods

### Human prostate cancer specimens

Prostate tissue samples were collected as part of an Institutional Review Board approved protocol at Henri Mondor Hospital in France (CPP 16169, French). Consent was obtained from all patients. In this cohort, 129 PCa tissue samples were collected, including 116 samples from radical prostatectomy of patients that did not receive prior hormone treatment at the hospital and 13 tissues collected by transurethral resection from Castrate-Resistant Prostate Cancer (CRPC) patients. Among these tissues, 9 HNPC specimens derived from normal peritumoral tissues were paired with tumoral tissues. All information is provided in [11]. The Cancer Genome Atlas (TCGA) cancer 2015 and 2018 data sets based on TCGA Research Network (http://cancergenome.nih.gov) were queried for mRNA expressions in prostate cancer using cBioportal and GTEx data using Xena platform [12].

### Immunohistochemistry analysis

The protocol was performed as previously described with minor modifications [11]. Tissues from Tissue MicroArray with patients from cohort described [11] were sectioned at 5 µm thickness, deparaffinized, and rehydrated. Antigen were unmasked by heat retrieval with pH 9 EDTA buffer for 15 min and endogenous peroxidase activity was inactivated with a 3% hydrogen peroxide solution for 10 min. TMA were then immuno-stained overnight at 4°C with anti-CDO1 antibody diluted in antibody diluent (Zytomed). Immuno-complexes were revealed using the anti-mouse Polink HRP mouse kit and the DAB substrate (Diagomics) with an incubation of the secondary mouse antibody for 30 min at room temperature. Tissues were then stained with hematoxylin and dehydrated. Slides were mounted using Eukitt medium. Protein expression was scored as null (0), weak (1), moderate (2), and strong (3) and the percentage of tumor cells stained was noted. The multiplication of the score and the percentage of stained tumor cells gave the quick score (between 0 and 300). Analysis was performed separately by two investigators (CC and VF).

### HTA2.0 and RNA sequencing data

Transcriptomic data from patient samples can be consulted from GSE200879 and GSE115414. The Cancer Genome Atlas cancer datasets were queried for mRNA expressions in prostate cancer using cBioportal and GTEx data using Xena platform 4.2.

For siCDO1 RNAseq, total RNA was extracted using RNeasy mini kit (Qiagen). RNA concentration and purity were tested using Nanodrop technology. Library preparation, capture, and sequencing analysis were performed by Novogene (UK). Messenger RNA were purified from total RNA using polyT oligo-attached magnetic beads, fragmented, and converted into first-strand cDNA using random hexamer primers, followed by second- strand synthesis incorporating dUTP to generate strand specific (directional) libraries. Libraries underwent end repair, Atailing, adapter ligation, size selection, amplification and purification, and RNA dosage were assessed by Qubit and realtime PCR for quantification, together with electrophoretic evaluation of fragment size distribution. Quantified libraries were pooled according to effective concentration and sequenced on Illumina platforms using paired-end 150 bp chemistry. Raw image files were converted to base-called reads using Illumina’s CASAVA pipeline, generating FASTQ files containing read sequences and associated base-quality scores. Raw reads were processed using inhouse scripts to remove adapter contamination, polyN sequences and lowquality reads, yielding highquality clean reads; standard quality metrics (Q20, Q30, GC content) were computed. Gene level read counts were obtained using featureCounts v1.5.0p3. Differential expression analyses were performed in R (v4.5.2) using DESeq2 (v1.50.2). Each siRNA targeting CDO1 (siCDO1A and siCDO1C) was compared independently to the corresponding siRNA control (siCtrl), and log2 fold changes were moderated using the lfcShrink procedure. The resulting Pvalues were adjusted using the Benjamini–Hochberg method to control the false discovery rate, and genes with an adjusted *P* value ≤ 0.05 were considered differentially expressed. Venn diagrams were generated to visualize the overlap between DEG sets from siCDO1A versus siCtrl and siCDO1C versus siCtrl. Variance-stabilizing transformation (vst) was applied to normalized counts, and genewise zscores were computed for heatmap representation. Functional enrichment analyses were performed using clusterProfiler (v4.18.4) for KEGG pathway enrichment and GO.db (v3.22.0) for Gene Ontology enrichment, based specifically on the subset of genes commonly differentially expressed in both siCDO1A and siCDO1C conditions. RNAseq data are available on the NCBI Gene Expression Omnibus platform under the number GSE326541

### Cell culture

VCaP cell line was obtained from ATCC (ATCC-CRL-2876, Manassas, VA). Cells were cultured in DMEM medium (Thermofischer) supplemented with 10% fetal bovine serum (Thermofischer), 50 IU/mL penicillin (Thermofischer), 100 µg/mL streptomycin (Thermofischer), and 2 nM DHT (Sigma). VCaP cells were cultivated at 37 °C, 5% CO_2_ under humidified atmosphere. The cells were tested monthly for the presence of mycoplasma using the kit Venor®GeM qEP - Mycoplasma qPCR detection kit (Minerva Biolabs).

### Small interfering RNA transfection

Cells were transfected using small interference RNA targeting CDO1 (siCDO1 A 106589, siCDO1 C 146014, Ambion®, Life Technologies) and AR (Eurofins). Briefly, 4.10^5^ or 1.10^4^ VCaP cells were plated in 6 well-plate or 96 well-plate culture dish respectively in complete medium and transfected with 400 pmol or 20 pmol respectively of siRNA/Lipofectamine® RNAiMax (Invitrogen, Life Technologies) mix diluted in optiMEM medium. After 48 h, cells were detached and used to perform RT-qPCR, proliferation, viability, apoptosis, migration assay, and formation of tumorsphere in low adherence conditions.

### Migration assay

In total, 48 h after siRNA transfection, 2.5 × 10^5^ VCaP cells were plated in 8 µm Transwell® in 1% FBS complete medium. Transwell® were added in a 24-well plate with 20% FBS complete medium allowing the cell migration through the membrane pores. After 24 h, membranes were washed twice with DPBS, scraped in order to remove the cells on the top and finally cells were fixed with cold methanol. The membranes were then stained with DAPI and cells were counted on ten fields for each well as described in [13].

### Cell growth assay

Cell viability was estimated by WST1 (Sigma, Roche) or MTT assays. WST1 reactive was added and absorbance was read at 450 nm after 2 h at 37 °C. For MTT assay, MTT reagent was added for 2 h at 37 °C then supernatant was removed and formazan crystals were dissolved in isopropanol while agitated for 30 min, and ultimately, absorbance was read at 550 nm.

### Prostatosphere formation in low adherence condition

Cells were plated in 96-well low adherence plate (1 × 10^3^ and 2 × 10^3^ cells/well) for tumorsphere formation using tumorsphere medium (DMEM F12; B27 supplement; 0.4% BSA; 10 ng/mL basic fibroblast growth factor (FGF), 20 ng/mL epidermial growth factor (EGF), 5 µg/mL insulin). Tumorspheres in 96-well low adherence plate were enabled to grow for 6 days and then pictures were captured using Olympus Cell A software (x100). Measuring the diameter and counting the number of spheres (>50 nm) were done using the Fiji Image J Software.

### Androgenic starvation in VCaP cells

Cells were seeded in 6-well plates (4 × 10^5^ cells/well) in DMEM medium supplemented with 10% FBS and 1% P/S. After 3 days, culture medium was washed three times and replaced with starvation medium (DMEM without red phenol, 10% glutamax, 10% charcoal serum) for 72 h. After 48 h of starvation, 2 nM or 10 nM of DHT or 10 µM of enzalutamide or apalutamide were added for a 6 or 24 h treatment. For the viability assays, cells were seeded in 96-well plates and transfected with siRNAs, 48 h after transfection, culture medium was removed and cells were washed three times and replaced with starvation medium (DMEM without red phenol, 10% glutamax, 10% charcoal stripped FBS) for 48 h and a MTT assay was performed.

### Apoptosis assay

Cells were transfected with siRNA as previously described. The assay was performed 48 h post-transfection following trypsinization and wash in phosphate buffered saline (PBS without Ca^2+^) at room temperature and using the Annexin V Apoptosis Detection Kit FITC (BD Biosciences). Annexin V-FITC and PI binding were detected with a MQ16 flow cytometer (Miltenyi). The flow cytometry results were analyzed by the FlowJo software to calculate the % of Annexin V and PI positive cells.

### Protein preparation and western blot analysis

Treated cells were lysed in RIPA buffer. Protein samples were then denaturated at 95 °C and migrated on 10% acrylamide/bisacrylamide gel. After transfer on a PVDF membrane (ThermoFisher Scientific), blocking was performed in 5% nonfat dry milk for 1 h. Membranes were then incubated overnight at 4 °C with primary antibodies (Supplementary Table 1). Membranes were washed in TBS-tween before incubation with the corresponding HRP-conjugated secondary antibody for 1 h. Immune complexes were detected by chemiluminescence detection with Pierce ECL western blotting substrate (ThermoFischer Scientific) using G:BOX systems (Syngene). The quantification was carried out with the software ImageJ.

### Quantitative real-time RT-qPCR

Total RNA extraction was performed using TRIzol Reagent and chloroform/isopronanol/ethanol method. Total RNA concentration was assessed using Nanodrop One. Retrotranscription was carried out by means of the High-capacity cDNA Reverse Transcription kit (Life Technologies). A total of 50 ng to 500 ng of RNA were used in a total reaction volume of 20 µL.

qPCR was performed using Maxima SYBR Green/ROX qPCR Master Mix on a StepOnePlus Real-Time PCR system (Applied Biosystems, USA). Five nanograms of cDNA were used in a final PCR volume of 20 µL. Final calculations were made according to the 2^-ΔΔCt^ method. The list of primers is available in Supplementary Table 2.

### ChiPseq analysis

Fastq of ChIP-seq with AR antibody from GSE148358 were downloaded from SRA (SRP255862). Low quality reads were removed with default parameters (phred quality < 15) using fastp 1.0.1 [14]. Reads were mapped using Bowtie2 2.5.5 [15] on hg19. BAM were processed with samtools 1.22.1 [16]. Blacklisted regions [17] were removed with bedtools v2.31.1 [18]. Peak calling was done using default parameters with Macs3 3.0.4 [19]. AR binding sites were identified with FIMO 5.0.5 [20] using motifs from JASPAR. Vizualisation was obtained using IGV [21].

### Statistical analyses

All statistical analyses were carried out using GraphPad software. For qPCR analysis with two groups, analysis of TMA, microarrays, TCGA, GDS1439, GSE35988 data with two groups, *t* test two-tailed was performed. For CDO1 Western blot and qPCR between different prostatic cells, qPCR for siRNA validation and analysis of genes regulated following transfection with siRNAs targeting CDO1 or AR, phenotypic tests (migration, proliferation, viability and non-adherent growth), ANOVA analysis was performed followed by Dunnett’s multiple comparison test. For microarray analysis with three or more groups, Western blot and qPCR in relation to androgen withdrawal trials or treatment with enzalutamide and apalutamide, analysis of data from the GDS2592 study, ANOVA analysis was performed followed by Turkey multiple comparison test. The correlation was analysed using Spearman’s test. Survival curves were compared using log-rank (Mantel-Cox) test. Values of *p* < 0.05 were considered significant. Results were expressed as mean ± SD of at least three determinations for each test from three independent experiments unless otherwise specified.

## Results

### CDO1 is under expressed in PCa relapsing patients

**Fig. 1:**
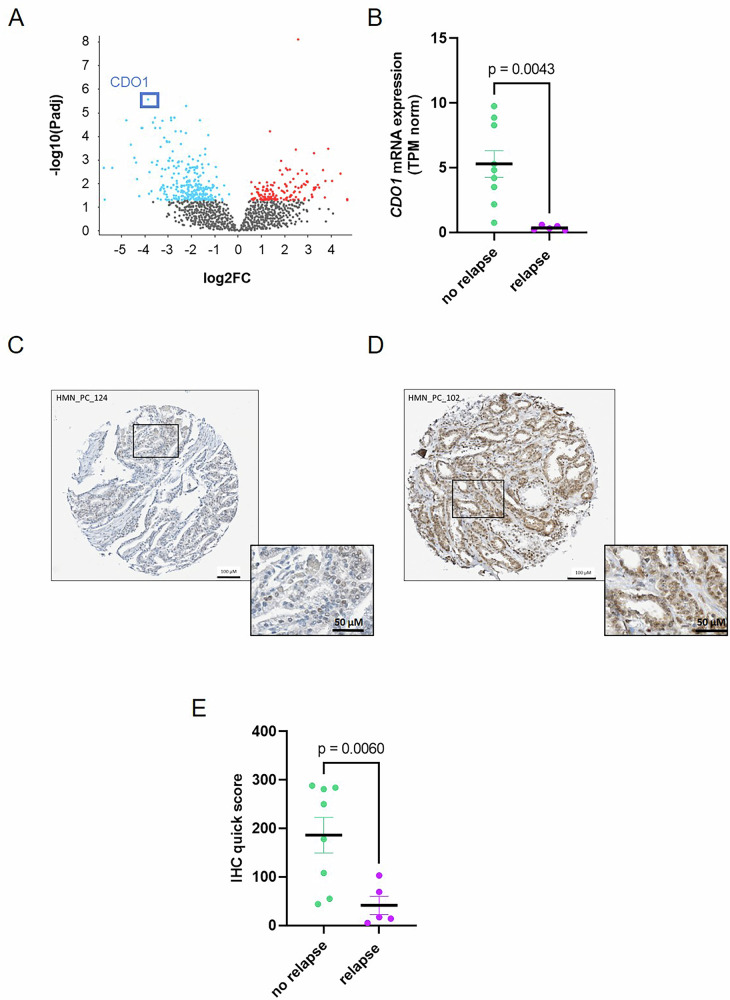
CDO1 is underexpressed in PCa relapsing patients. Analysis from GSE 115414 cohort (8 free of relapse and 5 relapsing patients. **A** Volcano plot of differential gene expression in the RNAseq data between relapsing patients as compared to no relapse patients. Blue square represents CDO1. **B** CDO1 expression by RNAseq. Immunohistochemistry targeting CDO1 protein was performed on tissue microarray from a relapsing patient (HMN_PC_124, **C**) and free of relapse patient (HMN_PC_102, **D**). Scale bars, 100 µm for TMA image and 50 µm for zoom. **E** Staining quantifications of CDO1 expression using quick scores defined by the intensity and the percentage of stained cells. Scores were blind analyzed by a pathologist.

In order to identify new markers that can predict tumor aggressiveness and the risk of relapse, we reanalysed GSE 115414 cohort data, considering recurrence status. This cohort includes 13 patients not treated prior to prostatectomy as described in Supplementary Table 3. We identified *CDO1* as part of the strongest under expressed genes in relapsing patient (Fig. 1A, B).

We then assessed the protein expression of CDO1 by IHC on tumor tissues of the same patients. CDO1 expression displayed mainly a nuclear localization, consistent with its attended localization. Its expression was highly decreased in patients with relapse (Fig. 1C) compared to patient with no relapse (Fig. 1D). CDO1 quantification using Quick Score, based on level intensity of the positive tumor cells, demonstrated a significant decrease in CDO1 expression for patients with relapse (Fig. 1E).

### Lower expression of CDO1 is associated with a poorer survival prognostic

**Fig. 2:**
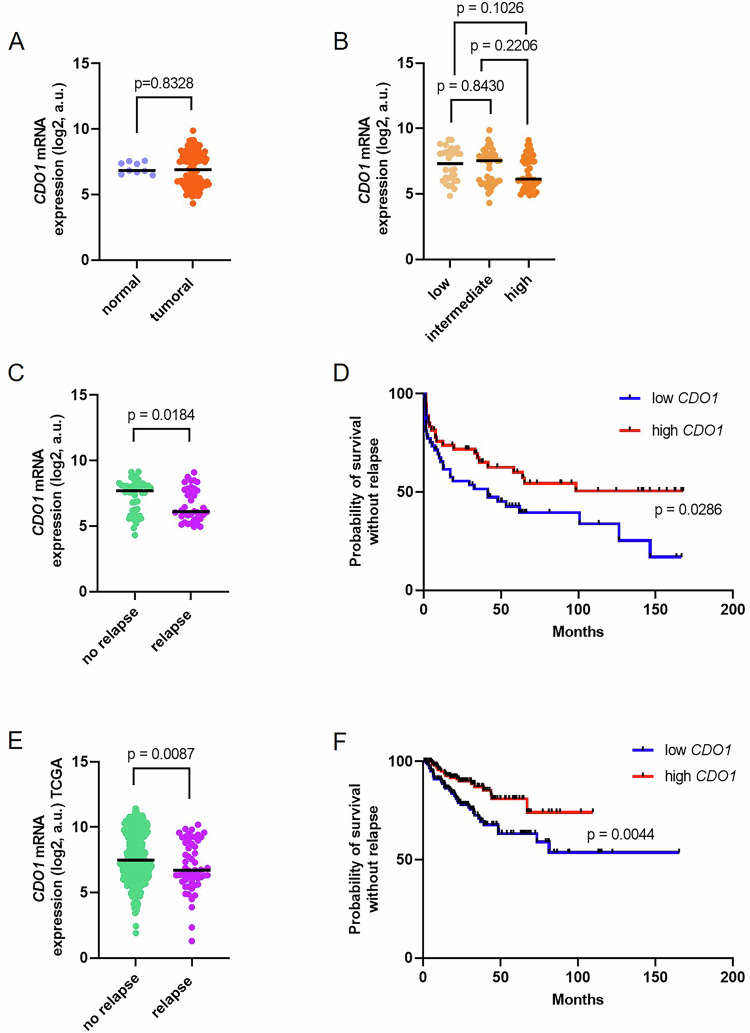
Lower expression of CDO1 is associated with poorer survival pronostic. mRNA expression of CDO1 in prostate tissues as measured by HTA2.0 (GSE200879) (**A**) in 9 normal tissues and 117 PCa (**B**) in 30 low risk, 44 intermediate and 43 high risk PCa, **C** in 52 relapse free PCa and 38 relapse PCa, **D** progression without relapse in GSE200879 in CDO1 low and CDO1 high patients (25% quartile), **E** gene expression of CDO1 in TCGA data using Xena platform. **F** progression without relapse in TCGA data in CDO1 low and CDO1 high patients (25% quartile).

To confirm if CDO1 could be a potential biomarker to identify relapsing forms of PCa, we analyzed *CDO1* expression in our GSE200879 cohort [11] which contains more tumor tissue than GSE11514 (Supplementary Table 4). No difference in expression could be detected between normal and tumor tissues (Fig. 2A). Similarly, no significant difference could be found between high-, intermediate- and low-grade tumors but with a decrease in expression with grade (Fig. 2B). In contrary, a significant decrease in *CDO1* expression was detected in tissues from patients who relapsed compared to patients without relapse (Fig. 2C). In an interesting way, patients with low *CDO1* expression have a lower relapse-free survival rate than patients with high *CDO1* expression. (Fig. 2D). In order to validate these results, mRNA *CDO1* expression was evaluated by the analysis of PRAD-TCGA data. This analysis showed a comparable significant decrease in *CDO1* mRNA expression in patients relapsing in comparison with the no relapse group (Fig. 2E). The low *CDO1* expression was also found to be associated with a worse survival rate without relapse (Fig. 2F). Altogether, these data suggest that lower expression of *CDO1* is associated with relapsing and a worse survival prognostic.

### CDO1 expression is positively regulated by methylation

**Fig. 3:**
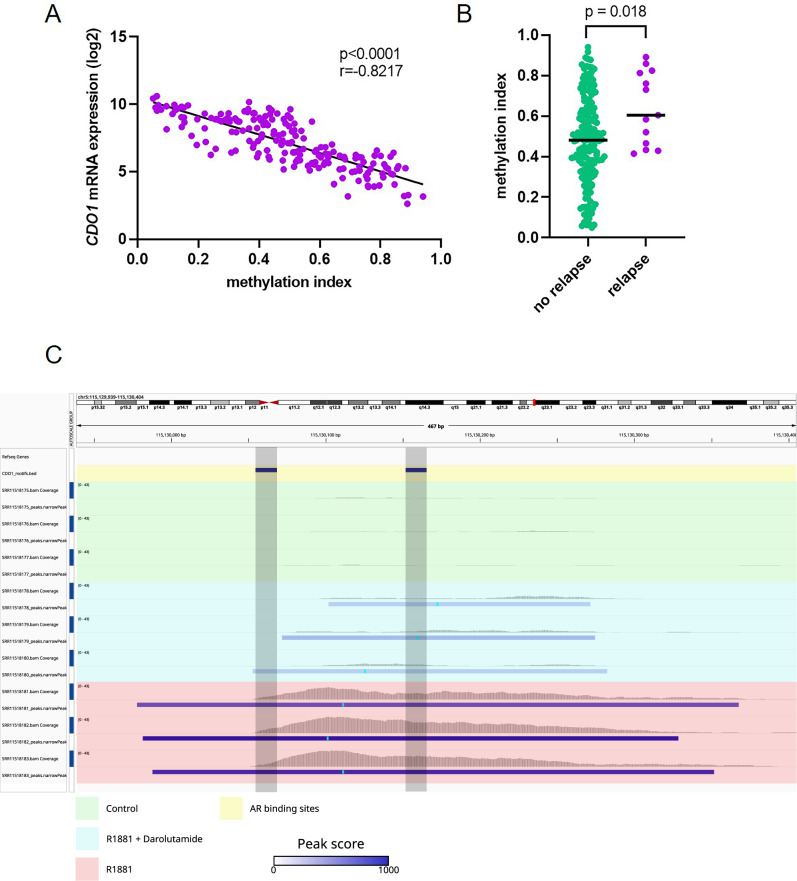
CDO1 expression is regulated by methylation. **A** Correlation between methylation profile at cg07644368 probe and CDO1 mRNA expression by RNAseq using Wanderer platform on TCGA PRAD patients. **B** Methylation profile at cg07644368 probe using Wanderer platform on TCGA PRAD patients. **C** Spatial representation of ChIP-seq enrichment for the androgen receptor (AR) (GSE148358) in regions upstream of the transcription start site of CDO1 gene.

Methylation of the *CDO1* promoter has been described in many cancers, including PCa. We therefore focused on potential methylation in PCa cases particularly in cases of relapse using the Wanderer platform for PRAD-TCGA. For cg07644368 probe, we observed a strong correlation between *CDO1* mRNA expression and methylation profile (Fig. 3A). By classifying these samples according to relapse or no relapse, hypermethylation was observed in this region in relapsing patients (Fig. 3B), confirming the role of methylation in *CDO1* regulation in recurrence.

### CDO1 expression is regulated by androgen pathway

PCa is known to be a hormone-dependent cancer in which many molecules involved in aggressiveness have been shown to be linked to regulation by the androgen pathway. We therefore sought to determine whether this was the case for CDO1 and whether it was controlled by androgen signaling. To support this hypothesis, we examined whether RNA-binding sites were present in the region upstream of the transcription start site (TSS) of the human *CDO1* gene and were thus able to identify putative AR-binding sites (Supplementary Fig. 1). To verify whether this binding was effective, we analyzed ChIP-seq data (GSE148358) from VCaP cells treated or untreated with R1881 and/or darolutamide (an AR antagonist). We observed enrichment approximately 10 kb upstream of the *CDO1* gene TSS at one of the putative binding sites in the R1881-treated condition, which decreased in the untreated and darolutamide-co-treated conditions (Supplementary Fig. 1, zoomed in on Fig. 3C). We therefore confirm that AR might bind upstream of the CDO1 gene to regulate it, and that this binding would depend on the presence of androgens.

Moreover, by reanalyzing data from GSD2569 transcriptome analysis [8], which focuses on genes regulated in vivo by androgens, we observed that *CDO1* expression was repressed after castration in mice and re-expressed when testosterone was injected into mice (Supplementary Fig. 2A). Under normal conditions in mice, *CDO1* would therefore be a target of androgens. To support this hypothesis in PCa, we have tested the effect of a modulation in the androgen receptor pathway in vitro using a prostate cancer cell model.

**Fig. 4:**
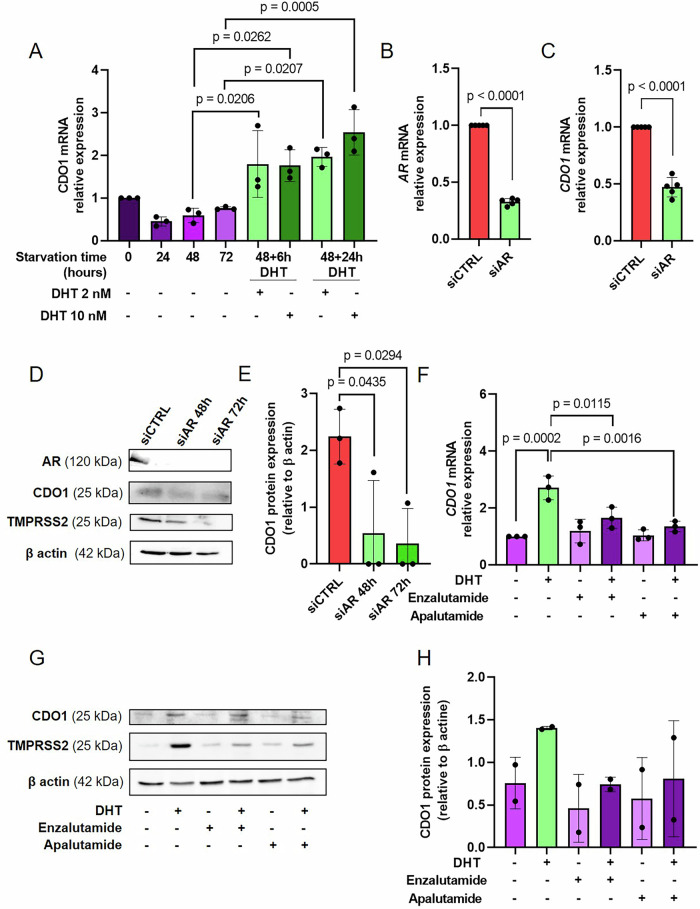
CDO1 expression is regulated by androgen signaling. **A** VCaP cells were starved for 0, 6, 24, 48 and 72 h then rechallenged with DHT (2 nM or 10 nM) after 48 h of starvation for 6 h or 24 h. mRNA levels of CDO1 were assessed by RT-qPCR, relative to RPLP0 expression. Each point represents the mean ± SD of three independent experiments performed in duplicate. **B**–**E** AR expression was disrupted using small interference RNA targeting AR in VCaP cells. mRNA levels performing RT-qPCR of AR (**B**) and CDO1 (**C**), relative to RPLP0 expression. Each point represents the mean ± SD of three independent experiments. **D** Western blot analysis of AR, TMPRSS2, and CDO1 expression in transfected VCaP cells. **E** Quantification of CDO1 protein expression relative to beta-actin. Each point represents the mean ± SD of three independent experiments. **F**–**H** VCaP cells were grown in androgen-free medium in the presence or absence of DHT (10 nM), enzalutamide (10 µM), and apalutamide (10 µM). **F** mRNA levels performing RT-qPCR, of and CDO1 were assessed, relative to RPLP0 expression. Each point represents the mean ± SD of three independant experiments. **G** Western blot analysis of CDO1 and TMPRSS2 in treated VCaP cells. **H** Quantification of CDO1 protein expression relative to beta-actin. Each point represents the mean ± SD of two independent experiments.

Among the different tested prostate tumor cell lines, the androgen sensitive cell line VCaP cells was the only one in which we observed CDO1 expression at both RNA and protein levels (Supplementary Fig. 2B, C, D). Concerning DHT effect, these cells were first cultivated in androgen deprived medium. After 48 h of deprivation, some cells have been treated with DHT (2 or 10 nM) or not for 6 h and 24 h. As expected, TMPRSS2 expression (positive control) was well reduced following androgen deprivation and overexpressed when DHT was added, whether at 2 or 10 nM (Supplementary Fig. 3A). In the same way, *CDO1* expression was reduced 1.5-fold at 48 h and increased twofold and 2.5-fold after 6 h and 24 h, respectively, with both concentrations (Fig. 4A), suggesting that *CDO1* expression is regulated by DHT.

We then tested the effect of AR silencing on CDO1 expression in VCaP cells, using small interference RNA. Transfection of VCaP cells with siRNA targeting AR (siAR) was performed during 48 h. AR expression was significantly decreased after 48 h (Fig. 4B and Supplementary Fig. 3B). Likewise, *TMPRSS2* mRNA expression was significantly decreased by 2.5-fold in the siAR condition (Supplementary Fig. 3C). A 2.5-fold decrease of *CDO1* mRNA expression was also observed (Fig. 4C). Again, the same results were observed at the protein level (Fig. 4D, E, Supplementary Fig. 3D).

To confirm the upregulation of CDO1 by androgen signaling, we decided to use two AR antagonists: enzalutamide and apalutamide. As described above, enzalutamide and apalutamide, by competing with DHT, lead to a decrease in *TMPRSS2* mRNA expression in VCaP cells (Supplementary Fig. 3E). With regard to CDO1, in the presence of enzalutamide or apalutamide, DHT no longer induced an increase in *CDO1* expression (Fig. 4F). The same result was observed at the protein level for TMPRSS2 and CDO1 (Fig. 4G, H, Supplementary Fig. 3F). Altogether, these results demonstrate the existence of an AR-CDO1 axis signaling in VCaP cells.

### Decrease in CDO1 transcriptional expression leads to increased tumor growth

**Fig. 5:**
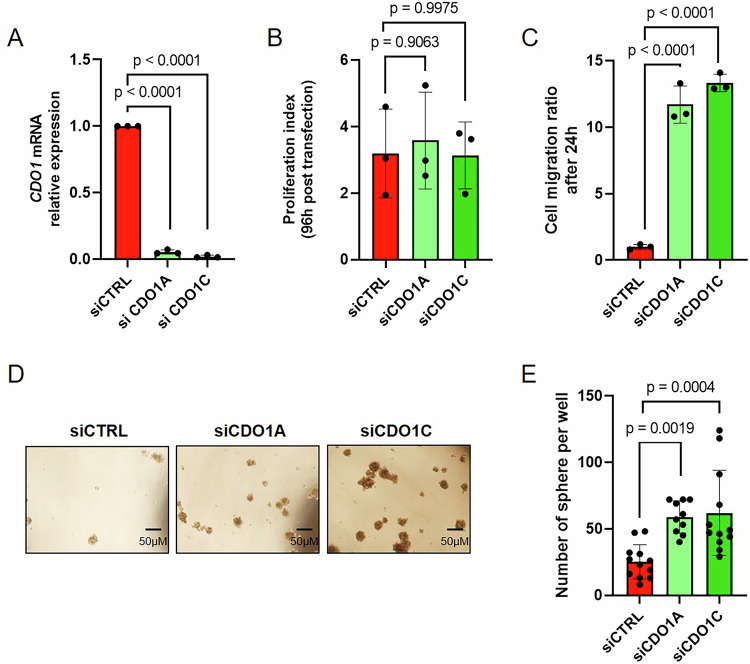
Decrease in CDO1 transcriptional expression leads to increased tumor growth. CDO1 expression has been disrupted using interfering RNA control or targeting CDO1 in VCaP cells for 48 h. **A** mRNA level of CDO1 performing RT-qPCR was assessed, relative to RPLP0 expression. Each point represents the mean ± SD of three independent experiments. **B** Cell migration of VCaP cells from 1% FBS medium to 20% FBS medium was analyzed using Transwell® assay. Each condition was made in triplicate. For each triplicate, cells undergoing migration were counted on ten fields. **C** Cell proliferation was measured using WST1 every 24 h during 4 days. Proliferation index at 96 h post transfection was calculated in triplicate. Each point represents the mean ± SD of three independent experiments. **D** Effect of CDO1 inhibition on tumorsphere formation observed by optique microscopy. **E** Determination of sphere forming. Each point represents a well counted from 4 independent experiments conducted in triplicate.

CDO1 implication in PCa tumor growth remains poorly described so far. Our previous in situ data showed that a decrease in CDO1 expression was found in patients with an aggressive phenotype. With the aim of understanding CDO1 role in tumor growth and aggressivity, VCaP cells were transfected with siRNA targeting CDO1 in order to decrease its expression. The silencing efficiency of siCDO1 A and C was confirmed with a decrease of 20-fold and 100-fold, respectively of mRNA expression and protein expression (Fig. 5A and Supplementary Fig. 4A, B). We were initially interested in the impact of CDO1 on cell growth and no significant difference of proliferation index was observed between siCDO1 A, C, and control conditions (Fig. 5B). To investigate the potential effect of CDO1 on cell migration, a Transwell® migration assay was carried out on the three conditions. A strong increase in migration of siCDO1 transfected cells was observed through the transwell membrane, suggesting a role for CDO1 in PCa cell migration (Fig. 5C). Another important factor in tumor aggressiveness is growth in non-adhesion condition, the impact of siCDO1 was evaluated on the formation of prostatospheres. The number of spheres was increased by threefold in the siCDO1 conditions A and C compared to the control condition. (Fig. 5D, E) suggesting an effect of CDO1 on PCa aggressiveness. In conclusion, loss of CDO1 appears to be involved in the migration and aggressiveness of PCa cells in vitro.

### Loss of CDO1 is associated with response to reticulum endoplasmic stress

**Fig. 6:**
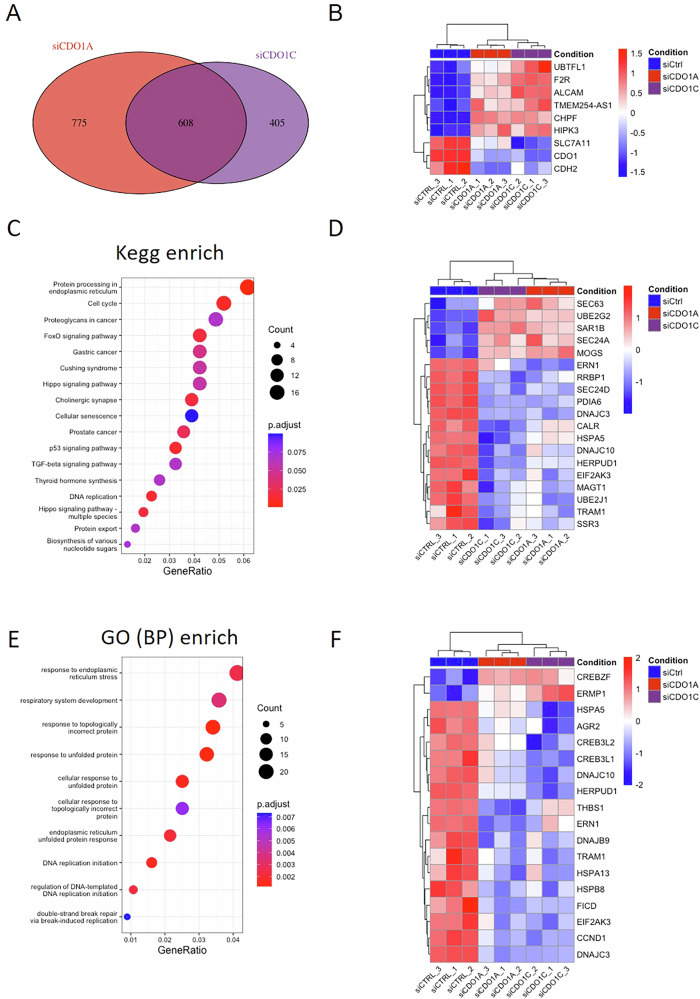
Loss of CDO1 is associated with response to reticulum endoplasmic stress. **A** Venn diagram showing the differentially expressed (DE) genes on VCaP transfected by siCDO1C and siCDO1A as compared to siCtrl. **B** Heatmap of DE genes FC > 1.3 qAdj<0.05. **C** KEGG analysis of the DE genes qAdj<0.05. **D** Heatmap of « protein processing in endoplasmic reticulum » for DE genes. **E** Gene Ontology of DE genes qAdj<0.05. **F** Heatmap of « response to endoplasmic reticulum stress » for DE genes.

**Fig. 7:**
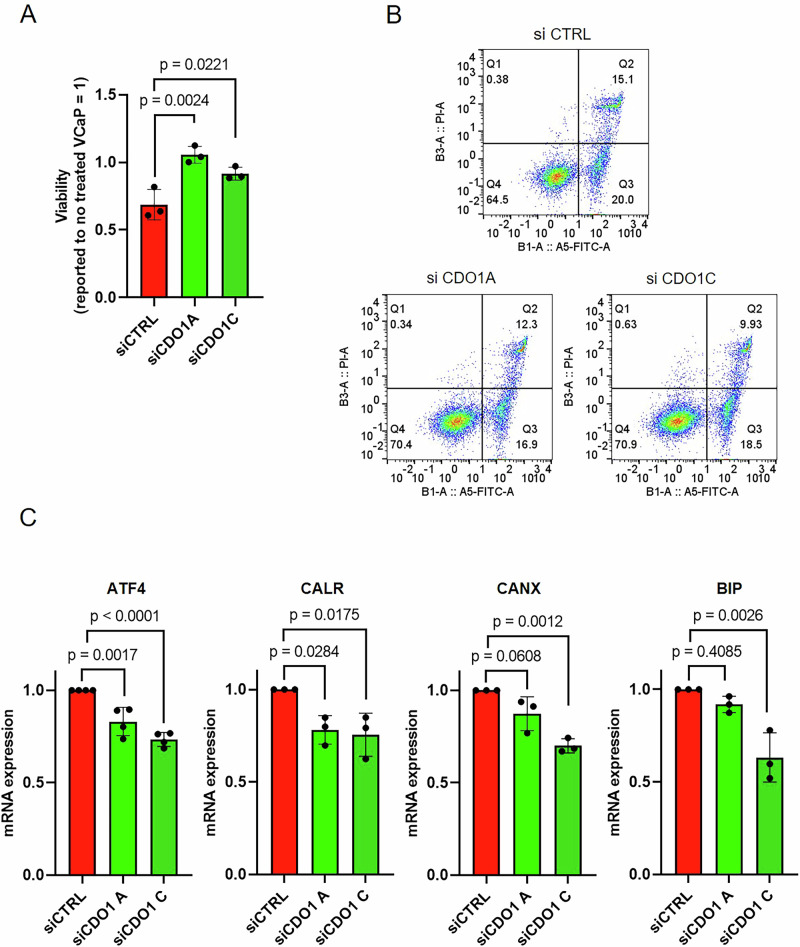
Loss of CDO1 is associated with response to reticulum endoplasmic stress. **A** Viability of VCaP cells inhibited for CDO1 expression after 48 h of androgen deprivation measured by MTT. Each point represents the mean ± SD of three independent experiments (each experiment contains a triplicate). **B** Apoptosis analysis of VCaP cells inhibited for CDO1 expression after 48 h of androgen deprivation measured by annexin V/PI test. Representative sample of two independent experiments. **C** CDO1 expression was disrupted using small interference RNA targeting AR in VCaP cells mRNA levels performing RT-qPCR of mRNA expression of ATF4, CALR, CANX, and BIP relative to RPLP0 expression. Each point represents the mean ± SD of three independent experiments.

To go further in our study, we investigated pathways associated with CDO1 expression decrease. RNAseq was performed on VCaP transfected with siRNA control or targeting CDO1. In this experiment, *CDO1* expression was significantly reduced by 82% and 88% with siCDO1 A and C, respectively (Supplementary Fig. 5A). Based on a qAdj-value of 0.05, 1383 genes were regulated between siCDO1 A transfected cells and siCtrl cells and 1013 between siCDO1 C transfected cells and siCtrl cells. Combining the results of both siRNA, 608 genes dysregulated have been described (Fig. 6A, Supplementary Fig. 5B, C). Only six RNAs displayed overexpression greater than 1.3 log-2 fold change after inhibition of CDO1 by either one of the two siRNAs: *F2R*, *ALCAM*, *TMEM254-AS1*, *HIPK3*, *UBTFL1*, and *CHPF* (Fig. 6B and Supplementary Fig. 6). Furthermore, only *SLC7A11* and *CDH2* appeared to be greatly reduced. Interestingly, it should be noted that *ALCAM*, *HIPK3* and *CHPF* have already been related to cell migration and aggressiveness in prostate or others cancers confirming the previous results. Given that few genes exhibit significant gene dysregulation, we searched for pathways that could be modulated by CDO1 inhibition. For this purpose, a KEGG enrichment analysis was carried out (Fig. 6C). Among all the pathways identified in this analysis, we can highlight in particular a modulation of the ‘protein processing in endoplasmic reticulum’ pathway (19 genes, Fig. 6D). To confirm these results, we also performed a GO enrichment analysis, showing enrichment on GO related to endoplasmic reticulum (ER) stress, unfolded protein and unfolded protein response (UPR) (Figs. 6E, F). CDO1 therefore appears to be involved in pathways related to the UPR and ER stress. As these different mechanisms and gene are often linked to cell death or viability mechanisms we therefore focused on the impact of CDO1 under androgen deprivation stress condition, described as inducer of ER stress [22]. Indeed, after 48 h without androgens, we observed an increase in viability of CDO1-inhibited VCaP (Fig. 7A) and an inhibition of apoptosis induced by androgen deprivation (Fig. 7B). In addition, we investigated the regulation by CDO1 of four genes involved in this pathway (CALR, CANX, ATF4, and BIP) and were able to demonstrate that these four genes were dysregulated following inhibition of *CDO1* by RNA interference (Fig. 7C). Altogether these results demonstrate a new role of CDO1 in response to reticulum endoplasmic stress.

## Discussion

In this study, we demonstrate that *CDO1* expression is significantly reduced in tumors at risk of biochemical recurrence and that low *CDO1* expression is associated with a higher risk of recurrence based on two independent cohorts. A decrease in expression therefore seems to indicate a more aggressive behavior of tumors. These results are consistent with the fact that *CDO1* expression is also decreased in PCa metastases. Indeed, reanalysis of the experimental data GDS1439 and GSE35988 showed lower CDO1 expression in metastases than in localized tumors (Supplementary Fig. 7A, B). However, contrary to Meller’s study [7] and what is known in other tumors [2], we did not find a decrease in CDO1 expression in tumors compared to normal tissue in our study. Nevertheless, our study only included nine patients with normal peritumoral tissue. Using TCGA data and based on previous study [7], it was demonstrated that the methylation level of *CDO1* promoter was significantly increased in PCa in particular in case of biochemical recurrence and we reported that it is related to a decreased of mRNA expression. We observe that only VCaP cells express CDO1 at significant levels. This is consistent with the findings of Meller et al., who reported higher promoter methylation in PC-3, LNCaP, 22RV1, and DU-145 cells (between 85% and 97%) than in VCaP cells (37%). To confirm this result, we tested CDO1 expression following demethylation with 5-aza-2’-deoxycytidine, we observed CDO1 re-expression in LNCaP, 22RV1, and DU-145 cells (Supplementary Fig. 8). These results suggest that methylation is a key factor in the repression of CDO1 independent of the AR pathway. Indeed, VCaPs have amplified AR, but this is also the case for 22RV1, which expresses low levels of CDO1. Since PCa is hormone-dependent at the localized stage, we considered the question of its regulation by androgens. We demonstrated that CDO1 was positively regulated by androgens in VCaP cells and that there are AR-binding sites upstream of the CDO1 TSS, whose activation depends on the presence of androgens. AR inhibition by AR antagonists or by siRNA resulted in decreased CDO1 expression. Our results are consistent with RNAseq analysis on VCaP cells cultured in serum-depleted medium with or without DHT (GSM243767 and GSM243765, respectively). A five-fold increase in CDO1 expression is observed in the presence of androgens. Similarly, and very interestingly, we observed a decrease in *CDO1* expression in CRPC tumors in our cohort compared to hormone-naive tumors (Supplementary Fig. 7C). This might seem surprising, as castration resistance is often associated with constitutive activation of the AR; however, at this stage, it is possible that the CDO1 promoter is hypermethylated, as described for 22RV1 cells (CRPC) compared to VCaP cells (HNPC) [5]. CDO1 hypermethylation is indeed described as a prognostic marker of aggressive disease or treatment resistance [17–19]. Furthermore, interestingly, it has been shown that AR binding to DNA can be attenuated by an oxidizing reagent; this appears to result from cross-linking of the cysteine residues in the DNA-binding domain of AR [21]. The alteration in redox homeostasis induced by CDO1 inhibition could therefore also affect AR binding at these targets. In conclusion, this is the first time that regulation of CDO1 expression by the androgenic pathway has been demonstrated in human cancers. However, regulation of *CDO1* by progestin 17α, 20β-dihydroxy-4-pregnen-3-one in the testis had already been demonstrated in Japanese eels [23].

Since the decrease in CDO1 appears to be linked to an aggressive tumor phenotype, we evaluated and confirmed its impact on cell proliferation and migration, and on non-adherent growth of VCaP cells. These results are consistent with those described in the literature for other cancers. Indeed, CDO1 overexpression in gastric cancer cells attenuates their proliferation [24]. In the same manner, forced expression of CDO1 in hepatocarcinoma cells [25] and esophageal squamous cancer cells [26] decreased tumor cell growth, migration, and invasion, and the ability of colony formation.

Transcriptomic analysis of VCaP cells inhibited for CDO1 expression also revealed dysregulation of genes already implicated in these mechanisms and linked to biochemical recurrence. In PCa, MMP-1-high/F2R-high expression and F2R high expression was described as an predictor for both unfavorable overall survival and biochemical recurrence-free survival [27, 28]. Concerning ALCAM/CD166, if Minner et al described that a high ALCAM/CD166 expressions levels were associated with a lower risk of a biochemical recurrence [29], on the contrary two others studies showed that higher levels of ALCAM in tissues or serum were predictive of shorter disease-free survival [30, 31]. ALCAM has also been related with metastasis forming through an effect on apoptosis, proliferation [32] and prostate sphere-forming cells regeneration[33].. Finally, CircHIPK3 has been described to induce cell proliferation, migration, and invasion of prostate cancer cells [34, 35].

Two of the genes dysregulated in cells inhibited for CDO1 expression are known to be involved in cell death. TMEM254-AS1, were correlated with the overall survival in ovarian cancer and linked to cuproptosis [36] and SLC7A11 is a molecule linked to ferroptosis and is a target of AR in PCa responsible to resistance to ferroptosis induced by anti-androgen treatment [37]. CDO1 is known to promote ferroptosis in gastric [8, 24], endometrial [38], hepatocarcinoma [39] and breast cancer cells [40]. However, KEGG and gene ontology analyses of our transcriptomic analyses did not allow us to identify any deregulation of the ferroptosis pathway.

The most surprising result concerns CDH2, but CDH2 is very poorly expressed in prostate cell lines and tumors. In many cancers, its role is linked to migration and invasion. In primary prostate cancer, few information is available, and even in the publication by Ku et al., there is a suggestion of a decrease in CDH2 in PCa [41]. Furthermore, an analysis of TCGA data seems to indicate that strong CDH2 expression is a good prognostic indicator for survival, which is consistent with our results. Finally, Figiel et al. demonstrated that CDH2 expression in tissues is not significantly associated with biochemical progression in localized PCa [42].

The pathways that appear to be significantly affected in our cancer cells are the endoplasmic reticulum stress pathway and protein unfolding response (UPR). When proteins misfold or accumulate in the ER, the cell activates these pathways to restore homeostasis or initiate cell death if the stress is too severe [29]. Our results seem to indicate that inhibition of CDO1 appears to deregulate the ER stress pathway and thus block death induced by this pathway. Several studies have developed an association between ER stress/UPR markers and PCa development in particular driven by androgens and AR signaling [43, 44]. IRE1α, PERK, and ATF6H are activated in PCa when cellular stress is detected in the ER to trigger UPR leading to survival effects [45], suggesting a critical function of ER stress in PCa progression. Moreover, five genes involved in the ER stress pathway had been identified as potential signatures of biochemical recurrence, but none were found in our analysis [46]. Interestingly, three of the genes (calnexin, calreticulin, HSP5A) found in our analysis have already been shown to be regulated by androgen signaling [47]. It therefore appears that inhibition of CDO1 would block part of the endoplasmic reticulum stress response particularly when this stress is induced by androgen deprivation.

CDO1 therefore appears to be a good marker for recurrent PCa prognosis and could be developed into a signature to improve treatment in prostate cancer patients with ISUP grade 3. Furthermore, our results highlight that CDO1 acts as anti-oncogene by influencing migration, and non-adherent growth, above all by regulating the ER stress pathway and the UPR response. This molecule therefore appears to be of interest not only as a marker but also as an interesting molecule to understand aggressivity mechanism in PCa. In addition, its downregulation and regulation in the CRPC and metastatic stages also warrant further study.

## Supplementary information

supplementary figure 1

supplementary figure 2

supplementary figure 3

supplementary figure 4

supplementary figure 5

supplementary figure 6

supplementary figure 7

supplementary figure 8

supplementary legends

supplementary materials

supplementary tables

## Data Availability

RNAseq data are available on the NCBI Gene Expression Omnibus platform under the number GSE326541.

## References

[1] Bray F, Laversanne M, Sung H, Ferlay J, Siegel RL, Soerjomataram I, et al. Global cancer statistics 2022: GLOBOCAN estimates of incidence and mortality worldwide for 36 cancers in 185 countries. CA: A Cancer J Clin. 2024;74:229–63. 10.3322/caac.21834. 10.3322/caac.21834 38572751

[2] Chen M, Zhu JY, Mu WJ, Guo L. Cysteine dioxygenase type 1 (CDO1): Its functional role in physiological and pathophysiological processes. Genes Dis. 2023;10:877–90. 10.1016/j.gendis.2021.12.023. 37396540 10.1016/j.gendis.2021.12.023 PMC10308199

[3] Heafield MT, Fearn S, Steventon GB, Waring RH, Williams AC, Sturman SG. Plasma cysteine and sulphate levels in patients with motor neurone, Parkinson’s and Alzheimer’s disease. Neurosci Lett. 1990;110:216–20. 10.1016/0304-3940(90)90814-p. 2325885 10.1016/0304-3940(90)90814-p

[4] Roman HB, Hirschberger LL, Krijt J, Valli A, Kožich V, Stipanuk MH. The cysteine dioxgenase knockout mouse: altered cysteine metabolism in nonhepatic tissues leads to excess H2S/HS− production and evidence of pancreatic and lung toxicity. Antioxid Redox Signal. 2013;19:1321–36. 10.1089/ars.2012.5010. 23350603 10.1089/ars.2012.5010 PMC3791055

[5] Ueki I, Roman HB, Valli A, Fieselmann K, Lam J, Peters R, et al. Knockout of the murine cysteine dioxygenase gene results in severe impairment in ability to synthesize taurine and an increased catabolism of cysteine to hydrogen sulfide. Am J Physiol Endocrinol Metab. 2011;301:E668–684. 10.1152/ajpendo.00151.2011. 21693692 10.1152/ajpendo.00151.2011 PMC3191547

[6] Brait M, Ling S, Nagpal JK, Chang X, Park HL, Lee J, et al. Cysteine dioxygenase 1 is a tumor suppressor gene silenced by promoter methylation in multiple human cancers. PLoS One. 2012;7:e44951 10.1371/journal.pone.0044951. 23028699 10.1371/journal.pone.0044951 PMC3459978

[7] Meller S, Zipfel L, Gevensleben H, Dietrich J, Ellinger J, Majores M, et al. CDO1 promoter methylation is associated with gene silencing and is a prognostic biomarker for biochemical recurrence-free survival in prostate cancer patients. Epigenetics. 2016;11:871–80. 10.1080/15592294.2016.1241931. 27689475 10.1080/15592294.2016.1241931 PMC5193493

[8] Hao S, Yu J, He W, Huang Q, Zhao Y, Liang B, et al. Cysteine dioxygenase 1 mediates erastin-induced ferroptosis in human gastric cancer cells. Neoplasia. 2017;19:1022–32. 10.1016/j.neo.2017.10.005. 29144989 10.1016/j.neo.2017.10.005 PMC5686465

[9] Jeschke J, O’Hagan HM, Zhang W, Vatapalli R, Calmon MF, Danilova L, et al. Frequent inactivation of cysteine dioxygenase type 1 contributes to survival of breast cancer cells and resistance to anthracyclines. Clin Cancer Res. 2013;19:12 10.1158/1078-0432.CCR-12-3751. 23630167 10.1158/1078-0432.CCR-12-3751 PMC3985391

[10] Jurkowska H, Roman HB, Hirschberger LL, Sasakura K, Nagano T, Hanaoka K, et al. Primary hepatocytes from mice lacking cysteine dioxygenase show increased cysteine concentrations and higher rates of metabolism of cysteine to hydrogen sulfide and thiosulfate. Amino Acids. 2014;46:1353–65. 10.1007/s00726-014-1700-8. 24609271 10.1007/s00726-014-1700-8 PMC4930650

[11] Firlej V, Soyeux P, Nourieh M, Huet E, Semprez F, Allory Y, et al. Overexpression of nucleolin and associated genes in prostate cancer. Int J Mol Sci. 2022;23:4491 10.3390/ijms23094491. 35562881 10.3390/ijms23094491 PMC9101690

[12] Goldman MJ, Craft B, Hastie M, Repečka K, McDade F, Kamath A, et al. Visualizing and interpreting cancer genomics data via the Xena platform. Nat Biotechnol. 2020;38:675–8. 10.1038/s41587-020-0546-8. 32444850 10.1038/s41587-020-0546-8 PMC7386072

[13] El-Sayed IY, Daher A, Destouches D, Firlej V, Kostallari E, Maillé P, et al. Extracellular vesicles released by mesenchymal-like prostate carcinoma cells modulate EMT state of recipient epithelial-like carcinoma cells through regulation of AR signalling. Cancer Lett. 2017;410:100–11. 10.1016/j.canlet.2017.09.010. 28935391 10.1016/j.canlet.2017.09.010

[14] Chen S. fastp 1.0: An ultra-fast all-round tool for FASTQ data quality control and preprocessing. Imeta. 2025;4:e70078. 10.1002/imt2.70078. 41112039 10.1002/imt2.70078 PMC12527978

[15] Langmead B, Wilks C, Antonescu V, Charles R. Scaling read aligners to hundreds of threads on general-purpose processors. Bioinformatics. 2019;35:421–32. 10.1093/bioinformatics/bty648. Hancock J, éditeur1 févr. 30020410 10.1093/bioinformatics/bty648 PMC6361242

[16] Danecek P, Bonfield JK, Liddle J, Marshall J, Ohan V, Pollard MO, et al. Twelve years of SAMtools and BCFtools. Gigascience. 2021;10:giab008. 10.1093/gigascience/giab008. 33590861 10.1093/gigascience/giab008 PMC7931819

[17] Amemiya HM, Kundaje A, Boyle AP. The ENCODE blacklist: identification of problematic regions of the genome. Sci Rep. 2019;9:9354. 10.1038/s41598-019-45839-z. 31249361 10.1038/s41598-019-45839-z PMC6597582

[18] Quinlan AR, Hall IM. BEDTools: a flexible suite of utilities for comparing genomic features. Bioinformatics. 2010;26:841–2. 10.1093/bioinformatics/btq033. 20110278 10.1093/bioinformatics/btq033 PMC2832824

[19] Zhang Y, Liu T, Meyer CA, Eeckhoute J, Johnson DS, Bernstein BE, et al. Model-based analysis of ChIP-Seq (MACS). Genome Biol. 2008;9:R137. 10.1186/gb-2008-9-9-r137. 18798982 10.1186/gb-2008-9-9-r137 PMC2592715

[20] Grant CE, Bailey TL, Noble WS. FIMO: scanning for occurrences of a given motif. Bioinformatics. 2011;27:1017–8. 10.1093/bioinformatics/btr064. 21330290 10.1093/bioinformatics/btr064 PMC3065696

[21] Robinson JT, Thorvaldsdóttir H, Winckler W, Guttman M, Lander ES, Getz G, et al. Integrative genomics viewer. Nat Biotechnol. 2011;29:24–6. 10.1038/nbt.1754. 21221095 10.1038/nbt.1754 PMC3346182

[22] Segawa T, Nau ME, Xu LL, Chilukuri RN, Makarem M, Zhang W, et al. Androgen-induced expression of endoplasmic reticulum (ER) stress response genes in prostate cancer cells. Oncogene. 2002;21:8749–58. 10.1038/sj.onc.1205992. 12483528 10.1038/sj.onc.1205992

[23] Higuchi M, Celino FT, Tamai A, Miura C, Miura T. The synthesis and role of taurine in the Japanese eel testis. Amino Acids. 2012;43:773–81. 10.1007/s00726-011-1128-3. 22045384 10.1007/s00726-011-1128-3

[24] Ma G, Zhao Z, Qu Y, Cai F, Liu S, Liang H, et al. Cysteine dioxygenase 1 attenuates the proliferation via inducing oxidative stress and integrated stress response in gastric cancer cells. Cell Death Discov. 2022;8:493 10.1038/s41420-022-01277-x. 36526626 10.1038/s41420-022-01277-x PMC9758200

[25] Choi Jil, Cho EH, Kim SB, Kim R, Kwon J, Park M, et al. Promoter methylation of cysteine dioxygenase type 1: gene silencing and tumorigenesis in hepatocellular carcinoma. Ann Hepatobiliary Pancreat Surg. 2017;21:181–7. 10.14701/ahbps.2017.21.4.181. 29264579 10.14701/ahbps.2017.21.4.181 PMC5736736

[26] Kwon J, Park M, Kim JH, Lee HW, Kang MC, Park JH. Epigenetic regulation of the novel tumor suppressor cysteine dioxygenase 1 in esophageal squamous cell carcinoma. Tumour Biol. 2015;36:7449–56. 10.1007/s13277-015-3443-x. 25903467 10.1007/s13277-015-3443-x

[27] Latil A, Bièche I, Chêne L, Laurendeau I, Berthon P, Cussenot O, et al. Gene expression profiling in clinically localized prostate cancer: a four-gene expression model predicts clinical behavior. Clin Cancer Res. 2003;9:5477–85. 14654526

[28] Wang J, Liu D, Zhou W, Wang M, Xia W, Tang Q. Prognostic value of matrix metalloprotease-1/protease-activated receptor-1 axis in patients with prostate cancer. Med Oncol. 2014;31:968. 10.1007/s12032-014-0968-6. 24805876 10.1007/s12032-014-0968-6

[29] Minner S, Kraetzig F, Tachezy M, Kilic E, Graefen M, Wilczak W, et al. Low activated leukocyte cell adhesion molecule expression is associated with advanced tumor stage and early prostate-specific antigen relapse in prostate cancer. Hum Pathol. 2011;42:1946–52. 10.1016/j.humpath.2011.02.017. 21683980 10.1016/j.humpath.2011.02.017

[30] Kristiansen G, Pilarsky C, Wissmann C, Stephan C, Weissbach L, Loy V, et al. ALCAM/CD166 is up-regulated in low-grade prostate cancer and progressively lost in high-grade lesions. Prostate. 2003;54:34–43. 10.1002/pros.10161. 12481253 10.1002/pros.10161

[31] Sanders AJ, Owen S, Morgan LD, Ruge F, Collins RJ, Ye L, et al. Importance of activated leukocyte cell adhesion molecule (ALCAM) in prostate cancer progression and metastatic dissemination. Oncotarget. 2019;10:6362–77. 10.18632/oncotarget.27279. 31695844 10.18632/oncotarget.27279 PMC6824871

[32] Hansen AG, Arnold SA, Jiang M, Palmer TD, Ketova T, Merkel A, et al. ALCAM/CD166 is a TGF-β-responsive marker and functional regulator of prostate cancer metastasis to bone. Cancer Res. 2014;74:1404–15. 10.1158/0008-5472.CAN-13-1296. 24385212 10.1158/0008-5472.CAN-13-1296 PMC4149913

[33] Jiao J, Hindoyan A, Wang S, Tran LM, Goldstein AS, Lawson D, et al. Identification of CD166 as a surface marker for enriching prostate stem/progenitor and cancer initiating cells. PLOS ONE. 2012;7:e42564 10.1371/journal.pone.0042564. 22880034 10.1371/journal.pone.0042564 PMC3411798

[34] Liu DC, Song LL, Li XZ, Liang Q, Zhang ZG, Han CH. Circular RNA circHIPK3 modulates prostate cancer progression via targeting miR-448/MTDH signalling. Clin Transl Oncol. 2021;23:2497–506. 10.1007/s12094-021-02650-5. 34142340 10.1007/s12094-021-02650-5

[35] Cai C, Zhi Y, Wang K, Zhang P, Ji Z, Xie C, et al. CircHIPK3 overexpression accelerates the proliferation and invasion of prostate cancer cells through regulating miRNA-338-3p. Onco Targets Ther. 2019;12:3363–72. 10.2147/OTT.S196931. 31118688 10.2147/OTT.S196931 PMC6503193

[36] Zhou M, Tang J, Huang G, Hong L. Prognostic significance and immune landscape of a cuproptosis-related LncRNA signature in ovarian cancer. Biomedicines. 2024;12:2640 10.3390/biomedicines12112640. 39595204 10.3390/biomedicines12112640 PMC11592286

[37] Sun R, Yan B, Li H, Ding D, Wang L, Pang J, et al. Androgen receptor variants confer castration resistance in prostate cancer by counteracting antiandrogen-induced ferroptosis. Cancer Res. 2023;83:3192–204. 10.1158/0008-5472.CAN-23-0285. 37527336 10.1158/0008-5472.CAN-23-0285 PMC10543964

[38] Wang R, Yu X, Ye H, Ao M, Xi M, Hou M. LncRNA FAM83H-AS1 inhibits ferroptosis of endometrial cancer by promoting DNMT1-mediated CDO1 promoter hypermethylation. J Biol Chem. 2024;300:107680. 10.1016/j.jbc.2024.107680. 39159808 10.1016/j.jbc.2024.107680 PMC11419805

[39] Zhang J, Yimamu M, Cheng Z, Ji J, Wu L, Feng J, et al. TRIM47-CDO1 axis dictates hepatocellular carcinoma progression by modulating ferroptotic cell death through the ubiquitin‒proteasome system. Free Radic Biol Med. 2024;219:31–48. 10.1016/j.freeradbiomed.2024.04.222. 38614226 10.1016/j.freeradbiomed.2024.04.222

[40] Yang J, Sun L, Liu XY, Huang C, Peng J, Zeng X, et al. Targeted demethylation of the CDO1 promoter based on CRISPR system inhibits the malignant potential of breast cancer cells. Clin Transl Med. 2023;13:e1423. 10.1002/ctm2.1423. 37740473 10.1002/ctm2.1423 PMC10517212

[41] Ku SC, Liu HL, Su CY, Yeh IJ, Yen MC, Anuraga G, et al. Comprehensive analysis of prognostic significance of cadherin (CDH) gene family in breast cancer. Aging (Albany NY). 2022;14:8498–567. 10.18632/aging.204357. 36315446 10.18632/aging.204357 PMC9648792

[42] Figiel S, Vasseur C, Bruyere F, Rozet F, Maheo K, Fromont G. Clinical significance of epithelial-mesenchymal transition markers in prostate cancer. Hum Pathol. 2017;61:26–32. 10.1016/j.humpath.2016.10.013. 27818287 10.1016/j.humpath.2016.10.013

[43] Storm M, Sheng X, Arnoldussen YJ, Saatcioglu F. Prostate cancer and the unfolded protein response. Oncotarget. 2016;7:54051–66. 10.18632/oncotarget.9912. 27303918 10.18632/oncotarget.9912 PMC5288241

[44] Farahani N, Alimohammadi M, Raei M, Nabavi N, Aref AR, Hushmandi K, et al. Exploring the dual role of endoplasmic reticulum stress in urological cancers: Implications for tumor progression and cell death interactions. J Cell Commun Signal. 2024;18:e12054. 10.1002/ccs3.12054. 39691874 10.1002/ccs3.12054 PMC11647052

[45] de la Calle CM, Shee K, Yang H, Lonergan PE, Nguyen HG. The endoplasmic reticulum stress response in prostate cancer. Nat Rev Urol. 2022;19:708–26. 10.1038/s41585-022-00649-3. 36168057 10.1038/s41585-022-00649-3

[46] Cen S, Jiang D, Lv D, Xu R, Hou J, Yang Z, et al. Comprehensive analysis of the biological functions of endoplasmic reticulum stress in prostate cancer. Front Endocrinol (Lausanne). 2023;14:1090277. 10.3389/fendo.2023.1090277. 36967783 10.3389/fendo.2023.1090277 PMC10036859

[47] Nantermet PV, Xu J, Yu Y, Hodor P, Holder D, Adamski S, et al. Identification of genetic pathways activated by the androgen receptor during the induction of proliferation in the ventral prostate gland. J Biol Chem. 2004;279:1310–22. 10.1074/jbc.M310206200. 14576152 10.1074/jbc.M310206200

