# Supplementary figures and images for: CDO1 is a new biomarker to discriminate aggressive forms of prostate cancer
Source: Oncogene. 2026 Jun 9;45(28):2795–807. doi: 10.1038/s41388-026-03842-5 (PMC13337485; doi:10.1038/s41388-026-03842-5)

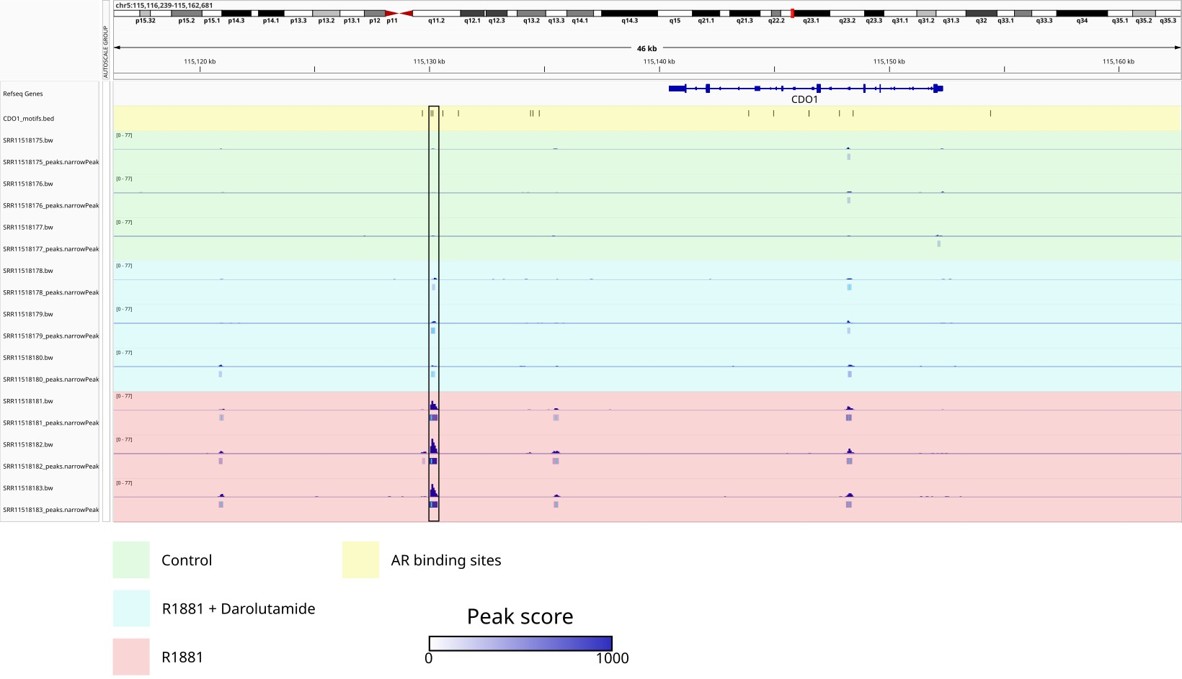

Supplement: Supplementary file 1 — supplementary figure 1 [file 41388_2026_3842_MOESM1_ESM.jpg]

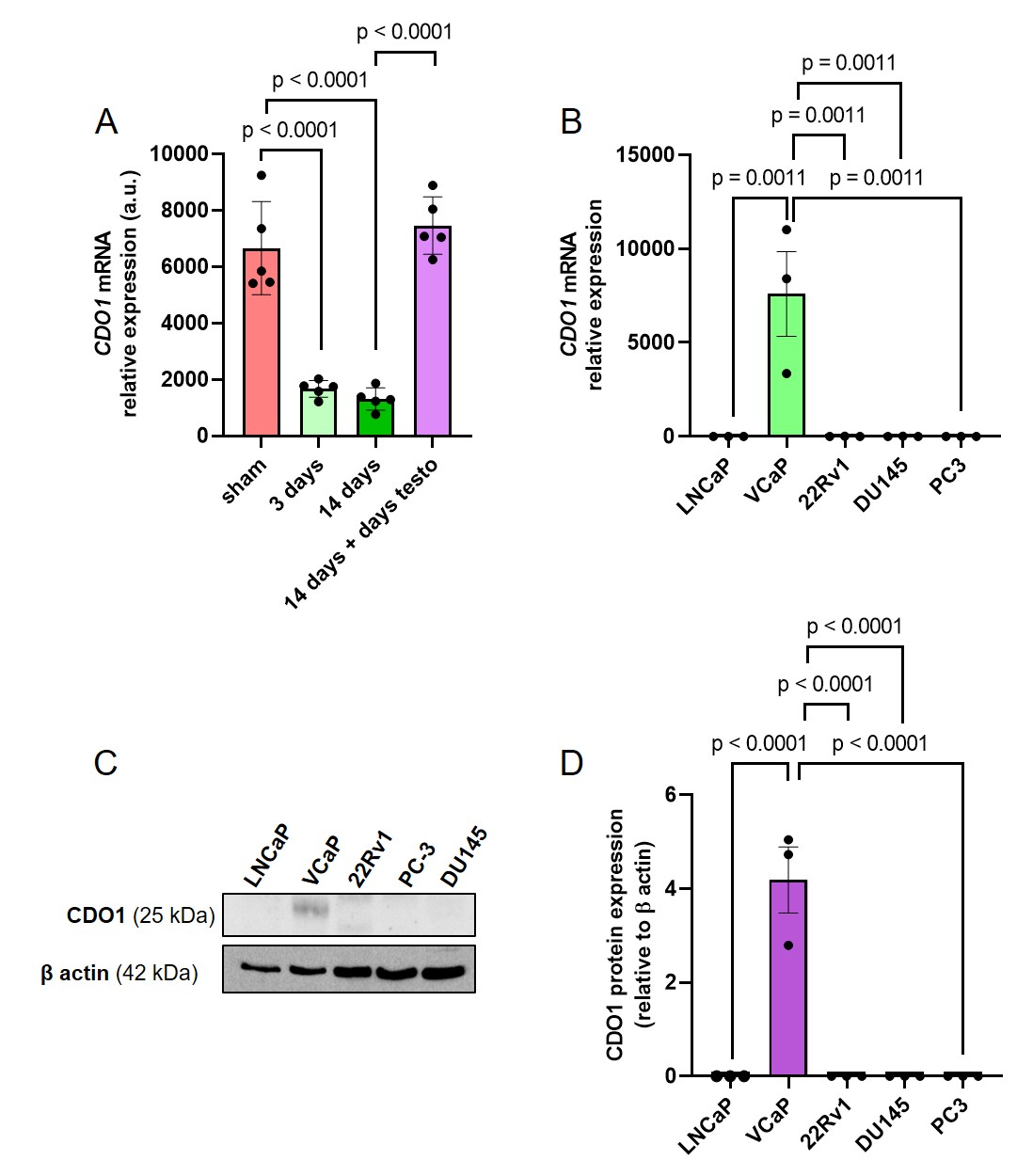

Supplement: Supplementary file 2 — supplementary figure 2 [file 41388_2026_3842_MOESM2_ESM.jpg]

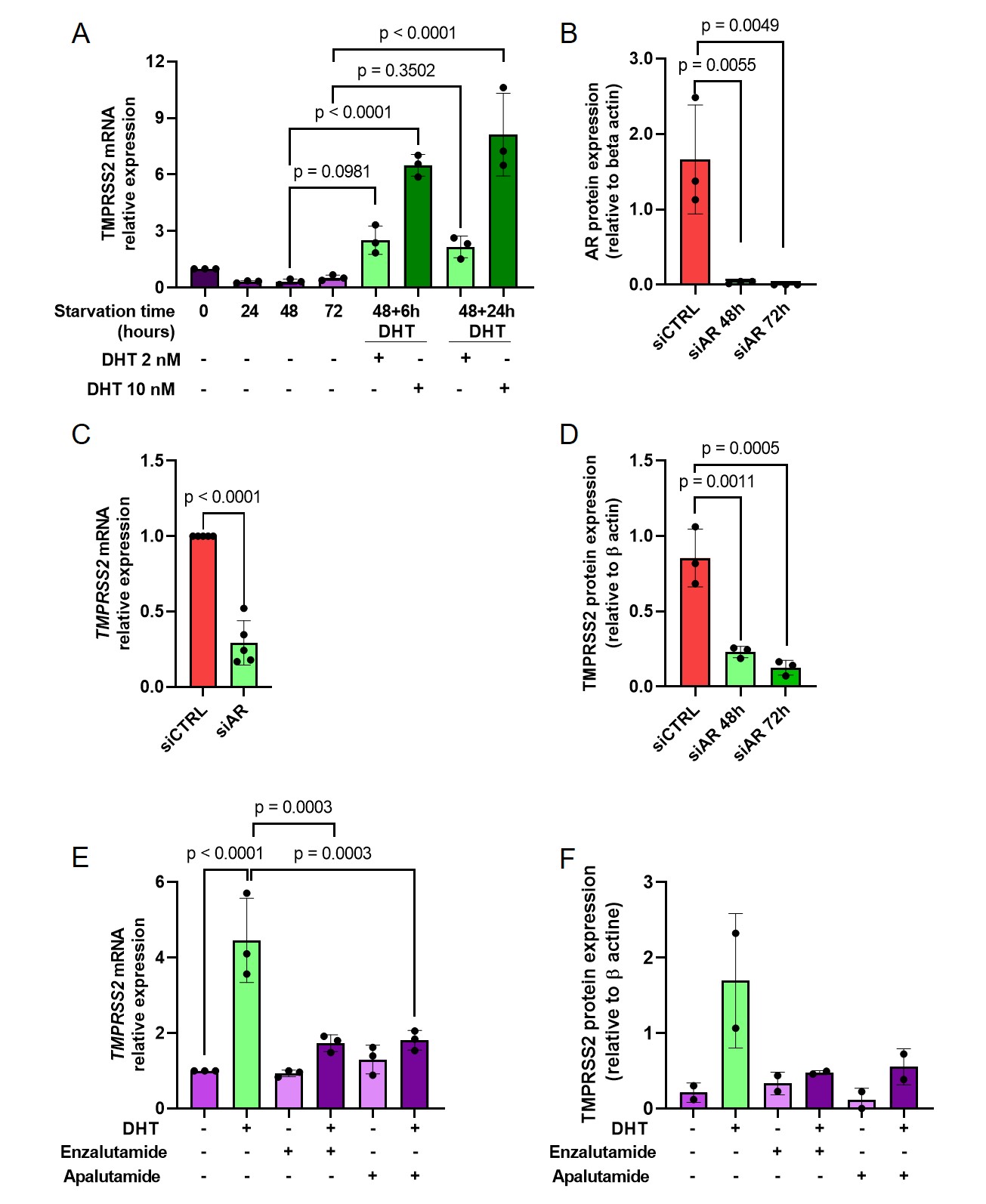

Supplement: Supplementary file 3 — supplementary figure 3 [file 41388_2026_3842_MOESM3_ESM.jpg]

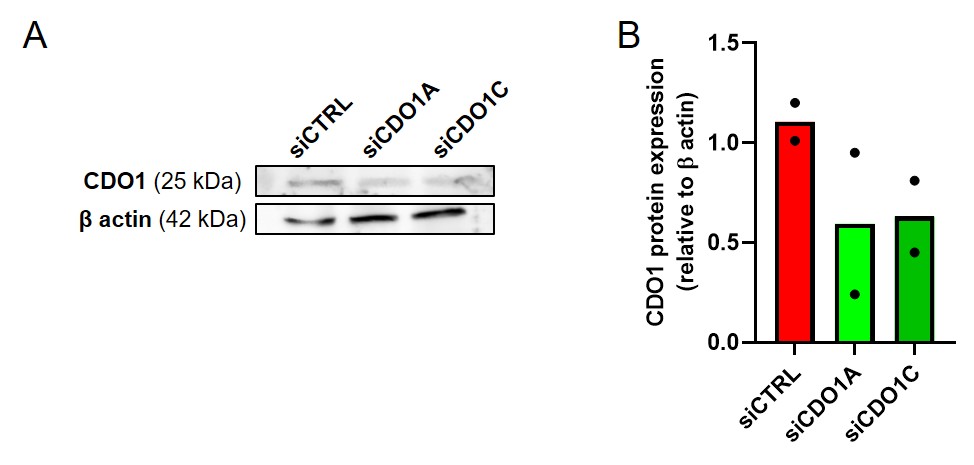

Supplement: Supplementary file 4 — supplementary figure 4 [file 41388_2026_3842_MOESM4_ESM.jpg]

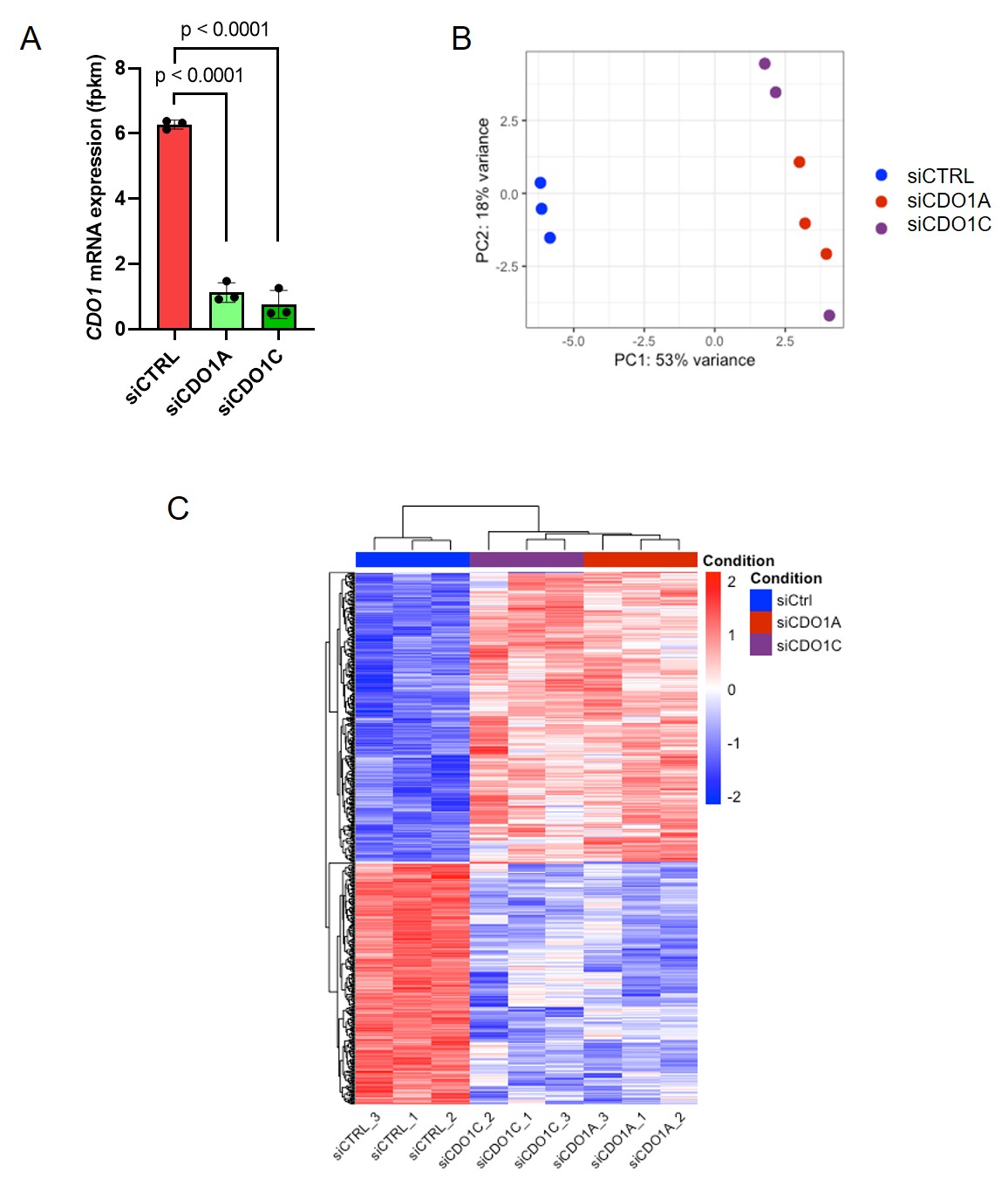

Supplement: Supplementary file 5 — supplementary figure 5 [file 41388_2026_3842_MOESM5_ESM.jpg]

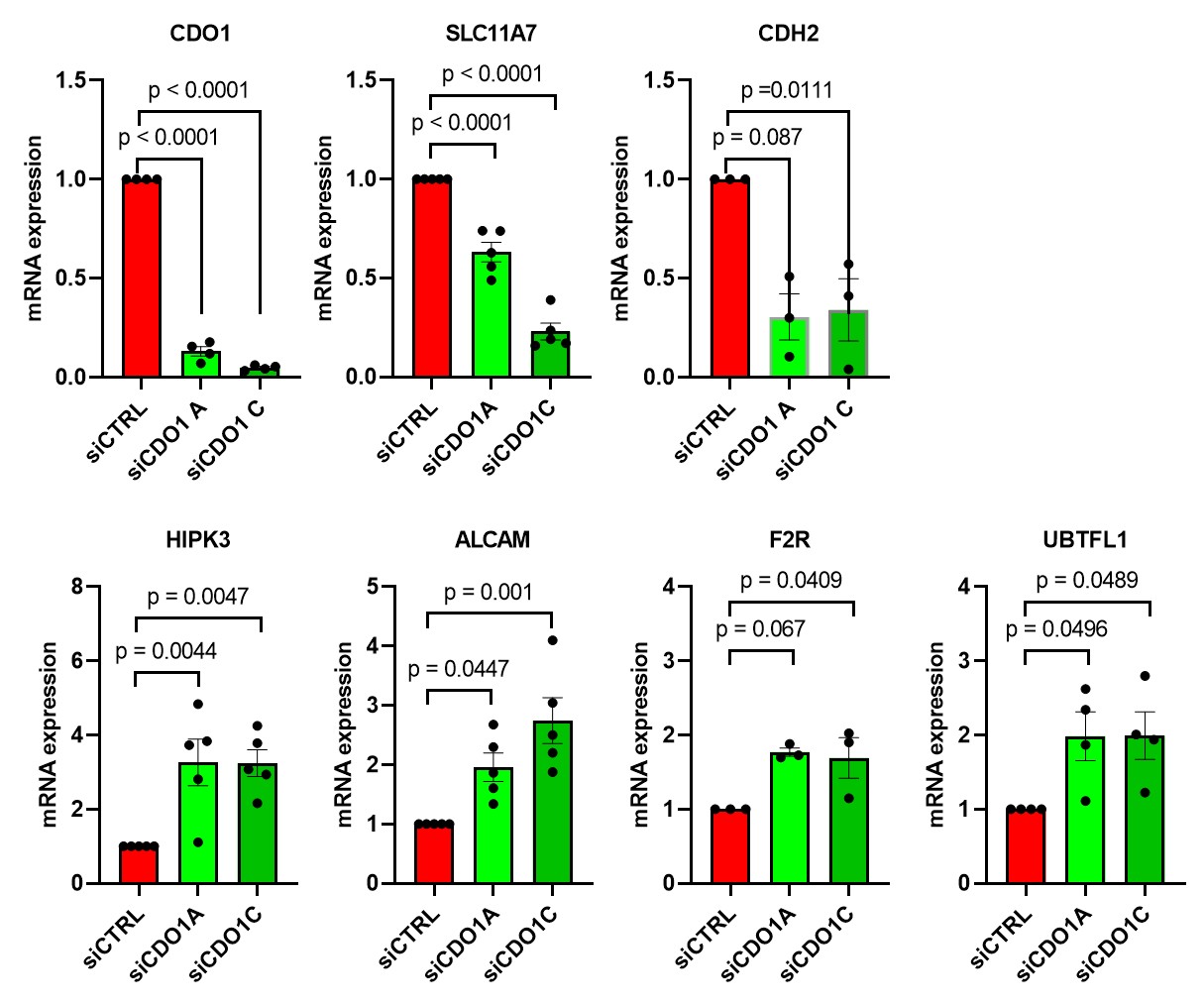

Supplement: Supplementary file 6 — supplementary figure 6 [file 41388_2026_3842_MOESM6_ESM.jpg]

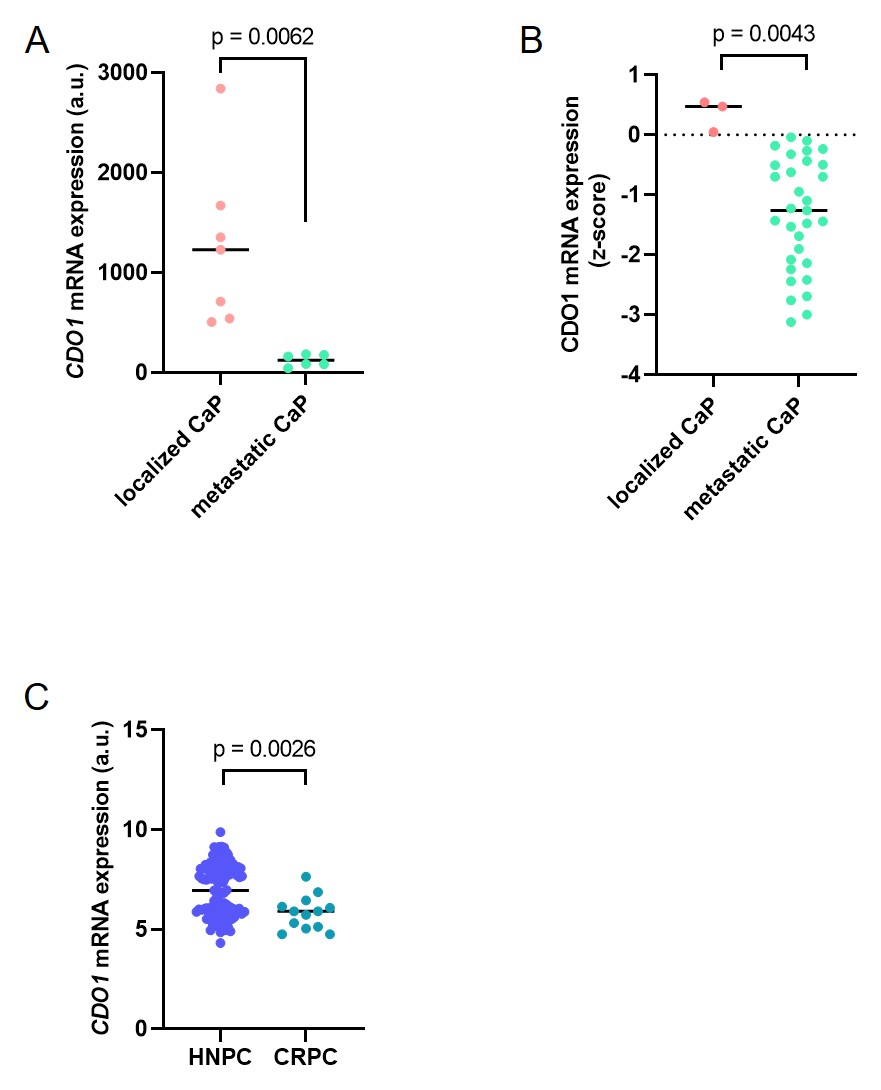

Supplement: Supplementary file 7 — supplementary figure 7 [file 41388_2026_3842_MOESM7_ESM.jpg]

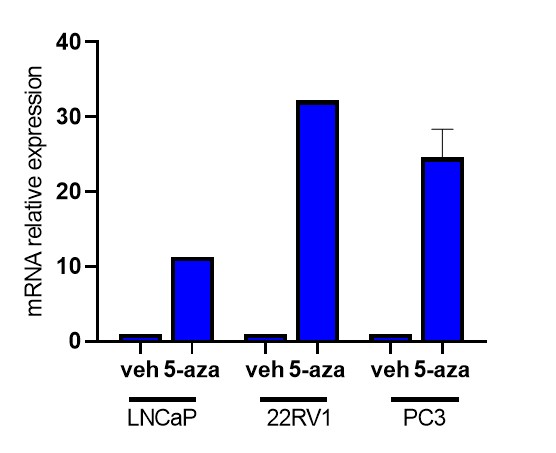

Supplement: Supplementary file 8 — supplementary figure 8 [file 41388_2026_3842_MOESM8_ESM.jpg]
