# Supplementary material for: CDO1 is a new biomarker to discriminate aggressive forms of prostate cancer
Source: Oncogene. 2026 Jun 9;45(28):2795–807. doi: 10.1038/s41388-026-03842-5 (PMC13337485; doi:10.1038/s41388-026-03842-5)
Supplement: Supplementary file 9 — supplementary legends [file 41388_2026_3842_MOESM9_ESM.docx]

**Supplementary legends**

**Supplementary Figure 1**: **AR can be set upstream of the TSS of CDO1**

Spatial representation of ChIP-seq enrichment for the androgen receptor (AR) (GSE148358) in regions upstream of the transcription start site of CDO1 gene.

**Supplementary figure 2: CDO1 is expressed in VCaP cells but not in other PCa cell lines**

(A) Expression of CDO1 mRNA from mice castrated at 3 and 14 days or traited 3 days with testostérone 14 days after castration (GSD2569). (B) mRNA expression of CDO1 in PCa cells lines measured by RT-qPCR. Results were normalized to RPLP0 by 2-ddCt. Relative quantities are expressed as mean±SD of three independent experiments. (C) Western blot analysis of CDO1 and beta-actin expression (D) Quantification of CDO1 protein expression relative to beta-actin.

**Supplementary Figure 3**: **CDO1 and TPRMSS2 expression is regulated by androgen signalling**

(A) VCaP cells were starved for 0, 6, 24, 48 and 72h then rechallenged with DHT (2nM or 10nM) after 48h of starvation for 6h or 24h. mRNA levels of TMPRSS2 were assessed by RT-qPCR, relative to RPLP0 expression. Each point represents the mean ± SD of three independent experiments performed in duplicate. (B, C, D) AR expression was disrupted using small interference RNA targeting AR in VCaP cells. (C) mRNA levels performing RT-qPCR of TMPRSS2 relative to RPLP0 expression. (B, D) Quantification of AR (B) and TMPRSS2 (D) protein expression relative to beta-actin. Statistics were performed using an ANOVA test followed by a Bonferroni post-test. (E, F) VCaP cells were grown in androgen-free medium in the presence or absence of DHT (10nM), enzalutamide (10µM) and apalutamide (10µM). (E) mRNA levels performing RT-qPCR, of TMPRSS2 were assessed, relative to RPLP0 expression. Each point represents the mean ± SD of three independant experiments. (F) Quantification of TMPRSS2 protein expression relative to beta-actin

**Supplementary Figure 4**: **CDO1 is underexpressed after siCDO1 transfection**

(A) Western blot analysis of CDO1 expression in siRNA targeting CDO1 transfected VCaP cells (B) Quantification of CDO1 protein expression relative to beta-actin. Each point represents the mean of two independent experiments

**Supplementary Figure 5**: **transcriptomic analysis of CDO1-inhibited VCaP**

CDO1 expression was disrupted using small interference RNA targeting AR in VCaP cells. (A) CDO1 mRNA expression by RNAseq (B) PCA of differentially expressed genes (C) Heatmap of DEGs gene (padj<0.05)

**Supplementary Figure 6**: **Effect of CDO1-inhibition on VCaP**

CDO1 expression was disrupted using small interference RNA targeting AR in VCaP cells. mRNA levels performing RT-qPCR of mRNA expression of CDO1, CDH2, FR2, HIPK3, ALCAM, SLC11A7 and UBTFL1 relative to RPLP0 expression. Each point represents the mean ± SD of three to five independent experiments

**Supplementary figure 7: CDO1 mRNA expression**

CDO1 mRNA expression in localized and metastatic PCa based on GDS1439 data (A) and GSE35988 (B), in HNPC and CRPC based on GSE200879 (C)

**Supplementary figure 8: CDO1 is regulated by methylation**

LNCaP, 22RV1 and PC3 cells were treated by 5-aza-2’-desoxycytidine during 48h. mRNA levels have been quantified by performing RT-qPCR of mRNA expression of CDO1. Each point represents one experiment.
