# Supplementary material for: CDO1 is a new biomarker to discriminate aggressive forms of prostate cancer
Source: Oncogene. 2026 Jun 9;45(28):2795–807. doi: 10.1038/s41388-026-03842-5 (PMC13337485; doi:10.1038/s41388-026-03842-5)
Supplement: Supplementary file 10 — supplementary materials [file 41388_2026_3842_MOESM10_ESM.docx]

**Supplementary materials**

**Cell culture**

LNCaP, 22RV1, DU145, PC-3 cell lines were obtained from ATCC (ATCC-CRL-1740, ATCC-CRL-2505, ATCC-HTB-81, ATCC-CRL-1435 respectively). Cells were cultured in RPMI medium (Thermofischer) supplemented with 10% fetal bovine serum (Thermofischer), 50 IU/mL penicillin (Thermofischer), 100 µg/mL streptomycin (Thermofischer). For LNCaP cells, 2nM DHT (Sigma) were added to the media. All cells were cultivated at 37°C, 5 % CO_2_ under humidified atmosphere. The cells were tested monthly for the presence of mycoplasma using the kit Venor®GeM qEP - Mycoplasma qPCR detection kit (Minerva Biolabs).

**5-aza-2’-desoxycytidine treatment**

5.10^4^ cells were seeded in 24-well plates culture dish. After 24 h of seeding, 5 µM of 5-aza-2’-desoxycytidine ((Sigma) were added for 72 h treatment. After 72 h, cells were lysed for RNA extraction and used to perform RT-qPCR.
