# Supplementary material for: CDO1 is a new biomarker to discriminate aggressive forms of prostate cancer
Source: Oncogene. 2026 Jun 9;45(28):2795–807. doi: 10.1038/s41388-026-03842-5 (PMC13337485; doi:10.1038/s41388-026-03842-5)
Supplement: Supplementary file 11 — supplementary tables [file 41388_2026_3842_MOESM11_ESM.docx]

Supplementary table 1

| **List of antibodies** |  |  |  |  |  |
| --- | --- | --- | --- | --- | --- |
|  |  |  |  |  |  |
| Antibody | Reference | Supplier | Species | Use | Dilution |
| CDO1 | 12589-1-AP | ThermoFischer | Rabbit | IHC, Western blot | 1/500 |
| TMPRSS2 | ab109131 | Abcam, Cambridge, UK | Rabbit | Western blot | 1/1000 |
| beta-actin | ab8226 | Abcam, Cambridge, UK | Mouse | Western blot | 1/1000 |
| secondary anti-rabbit IgG | #7074 | Cell signaling |  | Western blot | 1/2000 |
| secondary anti-mouse IgG | #7076 | Cell signaling |  | Western blot | 1/2000 |

Supplementary table 2

| **List of primers** | | |  | |
| --- | --- | --- | --- | --- |
|  |  | |  | |
|  | forward | reverse | |  |
| **CDO1** | TGGGGTGAAGGACATGGCAG | GCCAATGGAATCATTGATGTAGGC | |  |
| **UBTFL1** | AGCGCATGGTAGAGATTGGCAG | CAGGTGACAAAGTCTTGAGCCAG | |  |
| **F2R** | AGCGCATGGTAGAGATTGGCAG | CAGGTGACAAAGTCTTGAGCCAG | |  |
| **ALCAM** | TCCAGAACACGATGAGGCAGAC | GTAGACGACACCAGCAACAAGG | |  |
| **HIPK3** | CTACAGATCCGACCAGGAGTTC | TGTGAACCAGCCACACTCTCAG | |  |
| **SLC7A11** | TCCTGCTTTGGCTCCATGAACG | AGAGGAGTGTGCTTGCGGACAT | |  |
| **CDH2** | CAACGGGGACTGCACAGATG | TGTTTGGCCTGGCGTTCTTT | |  |
| **TMPRSS2** | AGGAGTGTACGGGAATGTGATGGT | GATTAGCCGTCTGCCCTCATTTGT | |  |
| **CANX** | AGCAGAGATGGCATGATGCT | GTTGAGGCTCATGATGGACA | |  |
| **CALR** | ATAAAGGGCTGCAGACAAGC | CCACAGTCGATATTCTGCTC | |  |
| **ATF4** | GGGACAGATTGGATGTTGGAGA | ACCCAACAGGGCATCCAAGT | |  |
| **BIP** | CATGGTTCTCACTAAAATGAAAG | GCTGGTACAGTAACAACTG | |  |

| Supplementary table 3:  **clinical features of the 13 patients from GSE115414** | | | | | | | | |  |
| --- | --- | --- | --- | --- | --- | --- | --- | --- | --- |
|  |  | | | |  |  |  |  |  |
| Patient | Stade | Gleason | Relapse |  | | | |  |  |
| HMN_PC_009 | pT3b | 8 (4 + 4) | no |  | | | |  |  |
| HMN_PC_077 | pT3a | 7 (3 + 4) | no |  | | | |  |  |
| HMN_PC_089 | pT2c | 7 (3 + 4) | no |  | | | |  |  |
| HMN_PC_102 | pT2a | 6 (3 + 3) | no |  | | | |  |  |
| HMN_PC_121 | pT3b | 8 (4 + 4) | yes |  | | | |  |  |
| HMN_PC_124 | pT3a | 8 (4 + 4) | yes |  | | | |  |  |
| HMN_PC_130 | pT4 | 6 (3 + 3) | yes |  | | | |  |  |
| HMN_PC_131 | pT3a | 8 (4 + 4) | yes |  | | | |  |  |
| HMN_PC_139 | pT3a | 6 (3 + 3) | no |  | | | |  |  |
| HMN_PC_148 | pT4 | 8 (4 + 4) | yes |  | | | |  |  |
| HMN_PC_167 | pT2c | 8 (4 + 4) | no |  | | | |  |  |
| HMN_PC_195 | pT3a | 8 (4 + 4) | no |  | | | |  |  |
| HMN_PC_228 | pT2a | 6 (3 + 3) | no |  | | | |  |  |
|  |  | | | |  |  |  |  |  |

Supplementary table 4 :

**Clinical features of patients from GSE200879**

|  |  |  |  |  |  |
| --- | --- | --- | --- | --- | --- |
|  |  |  |  |  |  |
| patient | Stade | Gleason | Risk | Relapse | Time of relapse (months) |
| HMN_PC_009 | pT3b | 8 (4 + 4) | high risk | no |  |
| HMN_PC_015 | pT2c | 7 (3 + 4) | low risk | no |  |
| HMN_PC_063 | pT3a | 8 (4 + 4) | high risk | yes | 5 |
| HMN_PC_065 | pT3a | 9 (4 + 5) | high risk | yes | 2 |
| HMN_PC_066 | pT3b | 7 (4 + 3) | high risk | yes | 2 |
| HMN_PC_067 | pT2a | 8 (4 + 4) | high risk | yes | 42 |
| HMN_PC_068 | pT3b | 9 (4 + 5) | high risk | yes | 8 |
| HMN_PC_070 | pT3a | 7 (4 + 3) | intermediate risk | yes | 7 |
| HMN_PC_071 | pT3b | 7 (4 + 3) | high risk | yes | 34 |
| HMN_PC_075 | pT3b | 7 (4 + 3) | high risk | yes | 101 |
| HMN_PC_076 | pT2c | 8 (4 + 4) | high risk | yes | 36 |
| HMN_PC_077 | pT3a | 7 (3 + 4) | intermediate risk | no |  |
| HMN_PC_077 | pT3a | 7 (4 + 3) | intermediate risk | yes | 1 |
| HMN_PC_078 | normal | normal | normal |  |  |
| HMN_PC_081 | pT3a | 7 (3 + 4) | intermediate risk | no |  |
| HMN_PC_082 | pT2c | 7 (3 + 4) | low risk | no |  |
| HMN_PC_083 | pT2a | 7 (3 + 4) | intermediate risk | no |  |
| HMN_PC_084 | pT2c | 7 (3 + 4) | low risk | yes |  |
| HMN_PC_085 | pT3b | 7 (4 + 3) | high risk | no |  |
| HMN_PC_086 | normal | normal |  |  |  |
| HMN_PC_087 | pT3a | 7 (4 + 3) | intermediate risk | no |  |
| HMN_PC_089 | pT2c | 7 (4 + 3) | intermediate risk | no |  |
| HMN_PC_092 | pT3a | 7 (4 + 3) | intermediate risk |  |  |
| HMN_PC_093 | normal | normal |  |  |  |
| HMN_PC_096 | pT2a | 7 (3 + 4) | low risk |  |  |
| HMN_PC_097 | pT2c | 7 (3 + 4) | low risk | no |  |
| HMN_PC_099 | pT2c | 6 (3 + 3) | low risk | no |  |
| HMN_PC_100 | pT3a | 7 (4 + 3) | intermediate risk | yes | 102 |
| HMN_PC_101 | pT2a | 6 (3 + 3) | low risk | yes | 54 |
| HMN_PC_102 | pT2a | 6 (3 + 3) | low risk | no |  |
| HMN_PC_103 | pT3a | 7 (4 + 3) | intermediate risk | no |  |
| HMN_PC_104 | pT3a | 7 (3 + 4) | intermediate risk | no |  |
| HMN_PC_105 | pT2c | 7 (3 + 4) | low risk | no |  |
| HMN_PC_106 | pT3a | 7 (3 + 4) | intermediate risk | no |  |
| HMN_PC_110 | pT3a | 7 (4 + 3) | intermediate risk | yes | 65 |
| HMN_PC_111 | pT2c | 6 (3 + 3) | low risk | no |  |
| HMN_PC_113 | pT2c | 7 (3 + 4) | low risk | no |  |
| HMN_PC_114 | pT2b | 6 (3 + 3) | low risk | no |  |
| HMN_PC_115 | pT2c | 6 (3 + 3) | low risk | no |  |
| HMN_PC_117 | pT3a | 6 (3 + 3) | intermediate risk | no |  |
| HMN_PC_118 | pT3a | 7 (4 + 3) | intermediate risk |  |  |
| HMN_PC_119 | pT2c | 7 (3 + 4) | low risk | no |  |
| HMN_PC_121 | pT3b | 8 (4 + 4) | high risk | yes | 1 |
| HMN_PC_123 | pT3b | 8 (4 + 4) | high risk | yes | 18 |
| HMN_PC_124 | pT3a | 8 (4 + 4) | high risk | yes | 2 |
| HMN_PC_125 | normal | normal |  |  |  |
| HMN_PC_126 | pT4 | 7 (4 + 3) | high risk | yes | 3 |
| HMN_PC_127 | pT3a | 8 (4 + 4) | high risk | yes | 2 |
| HMN_PC_128 | pT3a | 7 (4 + 3) | intermediate risk | yes | 8 |
| HMN_PC_130 | pT4 | 6 (3 + 3) | high risk | yes | 6 |
| HMN_PC_131 | pT3a | 8 (4 + 4) | high risk | yes | 18 |
| HMN_PC_132 | normal | normal |  |  |  |
| HMN_PC_133 | pT3a | 7 (3 + 4) | intermediate risk | yes | 42 |
| HMN_PC_134 | pT3a | 8 (4 + 4) | high risk | no |  |
| HMN_PC_135 | pT3a | 7 (4 + 3) | intermediate risk | yes | 4 |
| HMN_PC_137 | pT3b | 9 (4 + 5) | high risk | yes | 2 |
| HMN_PC_138 | pT2c | 7 (3 + 4) | low risk | yes | 3 |
| HMN_PC_139 | pT3a | 6 (3 + 3) | intermediate risk | no |  |
| HMN_PC_140 | pT3b | 7 (4 + 3) | high risk | yes | 42 |
| HMN_PC_141 | pT3a | 8 (4 + 4) | high risk | yes | 3 |
| HMN_PC_142 | pT3a | 8 (4 + 4) | high risk | yes | 13 |
| HMN_PC_143 | pT3b | 8 (4 + 4) | high risk | yes | 1 |
| HMN_PC_145 | pT3b | 9 (4 + 5) | high risk | yes | 31 |
| HMN_PC_146 | pT3b | 7 (4 + 3) | high risk | yes | 20 |
| HMN_PC_147 | pT2c | 7 (3 + 4) | low risk | yes | 1 |
| HMN_PC_148 | pT4 | 8 (4 + 4) | high risk | yes | 1 |
| HMN_PC_149 | normal | normal |  |  |  |
| HMN_PC_152 | pT3a | 7 (3 + 4) | intermediate risk | ? |  |
| HMN_PC_156 | pT2c | 6 (3 + 3) | low risk | no |  |
| HMN_PC_157 | pT3b | 7 (4 + 3) | high risk | no |  |
| HMN_PC_158 | pT2c | 7 (3 + 4) | low risk | no |  |
| HMN_PC_159 | pT3a | 7 (3 + 4) | intermediate risk | no |  |
| HMN_PC_160 | pT3a | 7 (3 + 4) | intermediate risk | no |  |
| HMN_PC_161 | pT2c | 6 (3 + 3) | low risk | no |  |
| HMN_PC_162 | pT3a | 7 (4 + 3) | intermediate risk | no |  |
| HMN_PC_164 | pT3a | 7 (4 + 3) | intermediate risk | no |  |
| HMN_PC_166 | pT3a | 7 (4 + 3) | intermediate risk | no |  |
| HMN_PC_167 | pT2c | 8 (4 + 4) | high risk | no |  |
| HMN_PC_168 | pT2a | 7 (4 + 3) | intermediate risk | yes | 63 |
| HMN_PC_169 | pT3a | 7 (4 + 3) | intermediate risk | no |  |
| HMN_PC_170 | pT3a | 7 (4 + 3) | intermediate risk | yes | 30 |
| HMN_PC_172 | pT3a | 8 (4 + 4) | high risk | yes | 13 |
| HMN_PC_173 | pT2c | 6 (3 + 3) | low risk | no |  |
| HMN_PC_174 | pT2c | 7 (3 + 4) | low risk | no |  |
| HMN_PC_195 | pT3a | 8 (4 + 4) | high risk | no |  |
| HMN_PC_196 | pT3a | 7 (4 + 3) | intermediate risk | no |  |
| HMN_PC_197 | pT3a | 7 (4 + 3) | intermediate risk | yes | 66 |
| HMN_PC_198 | pT3a | 7 (4 + 3) | intermediate risk | yes | 49 |
| HMN_PC_199 | pT3b | 7 (4 + 3) | high risk | yes | 59 |
| HMN_PC_200 | pT3a | 6 (3 + 3) | intermediate risk | no |  |
| HMN_PC_202 | normal | normal |  |  |  |
| HMN_PC_203 | normal | normal |  |  |  |
| HMN_PC_204 | pT2c | 7 (3 + 4) | low risk | no |  |
| HMN_PC_205 | normal | normal |  |  |  |
| HMN_PC_206 | pT2b | 6 (3 + 3) | low risk | no |  |
| HMN_PC_207 | pT3a | 6 (3 + 3) | intermediate risk | no |  |
| HMN_PC_208 | pT2c | 6 (3 + 3) | low risk | yes | 12 |
| HMN_PC_209 | pT3a | 7 (3 + 4) | intermediate risk | yes | 1 |
| HMN_PC_216 | pT3b | 9 (5 + 4) | high risk | yes | 1 |
| HMN_PC_217 | pT3b | 7 (4 + 3) | high risk | yes | 8 |
| HMN_PC_218 | pT3a | 7 (4 + 3) | intermediate risk | yes | 19 |
| HMN_PC_219 | pT3a | 8 (4 + 4) | high risk | yes | 1 |
| HMN_PC_220 | pT4 | 8 (4 + 4) | high risk | yes | 1 |
| HMN_PC_221 | pT3b | 7 (4 + 3) | high risk | yes | 10 |
| HMN_PC_222 | pT2c | 8 (3 + 5) | high risk | no |  |
| HMN_PC_223 | pT4 | 8 (4 + 4) | high risk | yes | 1 |
| HMN_PC_224 | pT3b | 8 (4 + 4) | high risk | yes | 11 |
| HMN_PC_225 | pT3a | 8 (4 + 4) | high risk | yes | 4 |
| HMN_PC_226 | pT3a | 8 (4 + 4) | high risk | yes | 9 |
| HMN_PC_227 | pT2c | 7 (4 + 3) | intermediate risk | no |  |
| HMN_PC_228 | pT2a | 6 (3 + 3) | low risk | no |  |
| HMN_PC_229 | pT3a | 7 (4 + 3) | intermediate risk | no |  |
| HMN_PC_232 | pT3a | 7 (4 + 3) | intermediate risk | yes | 33 |
| HMN_PC_233 | pT2c | 7 (3 + 4) | low risk | no |  |
| HMN_PC_234 | pT3a | 8 (4 + 4) | high risk | yes | 1 |
| HMN_PC_235 | pT2c | 6 (3 + 3) | low risk | no |  |
| HMN_PC_236 | pT3a | 7 (4 + 3) | intermediate risk | yes | 100 |
| HMN_PC_239 | pT2c | 6 (3 + 3) | low risk | no |  |
| HMN_PC_241 | pT3a | 7 (4 + 3) | intermediate risk | no |  |
| HMN_PC_242 | pT3a | 7 (3 + 4) | intermediate risk | no |  |
| HMN_PC_243 | pT3a | 7 (4 + 3) | intermediate risk | no |  |
| HMN_PC_244 | pT4 | 8 (4 + 4) | high risk | yes | 1 |
| HMN_PC_245 | pT3a | 7 (4 + 3) | intermediate risk | yes | 35 |
| HMN_PC_246 | pT3a | 7 (4 + 3) | intermediate risk | no |  |
| HMN_PC_247 | pT2c | 6 (3 + 3) | low risk | no |  |
